# Ensemble description of the intrinsically disordered N-terminal domain of the Nipah virus P/V protein from combined NMR and SAXS

**DOI:** 10.1038/s41598-020-76522-3

**Published:** 2020-11-11

**Authors:** Marco Schiavina, Edoardo Salladini, Maria Grazia Murrali, Giancarlo Tria, Isabella C. Felli, Roberta Pierattelli, Sonia Longhi

**Affiliations:** 1 Magnetic Resonance Center (CERM), University of Florence, Via Luigi Sacconi 6, 50019 Sesto Fiorentino, Italy; 2 Department of Chemistry “Ugo Schiff”, University of Florence, Via della Lastruccia 3-13, 50019 Sesto Fiorentino, Italy; 3 Florence Center for Electron Nanoscopy (FloCEN), University of Florence, Via della Lastruccia 3-13, 50019 Sesto Fiorentino, Italy; 4 Lab. Architecture et Fonction des Macromolécules Biologiques (AFMB), UMR 7257, Aix-Marseille University and CNRS, 163 Avenue de Luminy, Case 932, Marseille, France

**Keywords:** Biochemistry, Biophysics, Structural biology

## Abstract

Using SAXS and NMR spectroscopy, we herein provide a high-resolution description of the intrinsically disordered N-terminal domain (PNT, aa 1–406) shared by the Nipah virus (NiV) phosphoprotein (P) and V protein, two key players in viral genome replication and in evasion of the host innate immune response, respectively. The use of multidimensional NMR spectroscopy allowed us to assign as much as 91% of the residues of this intrinsically disordered domain whose size constitutes a technical challenge for NMR studies. Chemical shifts and nuclear relaxation measurements provide the picture of a highly flexible protein. The combination of SAXS and NMR information enabled the description of the conformational ensemble of the protein in solution. The present results, beyond providing an overall description of the conformational behavior of this intrinsically disordered region, also constitute an asset for obtaining atomistic information in future interaction studies with viral and/or cellular partners. The present study can thus be regarded as the starting point towards the design of inhibitors that by targeting crucial protein–protein interactions involving PNT might be instrumental to combat this deadly virus.

## Introduction

The Nipah virus (NiV), together with its close relative Hendra virus (HeV), is a zoonotic paramyxovirus responsible for severe encephalitis in humans. The NiV and HeV have been classified in the *Henipavirus* genus^1^ that also comprises the later on discovered Cedar virus (CedV)^2^. Because of their high pathogenic power, broad host range, high interspecies transmission and lack of therapeutics and vaccines, henipaviruses are classified as bio-security level 4 (BSL-4) pathogens and are considered as potential bio-terrorism agents.

The genome of henipaviruses is made of a non-segmented, single-stranded RNA molecule of negative polarity that is encapsidated by a regular array of nucleoprotein (N) monomers to form a helical nucleocapsid. This N:RNA complex, and not naked RNA, is the substrate used by the viral polymerase for both transcription and replication. The viral polymerase is a complex consisting of the large (L) protein, which bears all the enzymatic activities, and the phosphoprotein (P). Through its interaction with both L and the nucleocapsid, the P protein acts as a tether and recruits L onto the N:RNA template. In addition, P also serves as a chaperon for both L^3^ and N in that it is required for proper folding/maturation of L and maintains N in a monomeric, RNA-free form^4^. Therefore P is a pivotal protein endowed with multiple functions critical for both transcription and replication.

**Figure 1:**
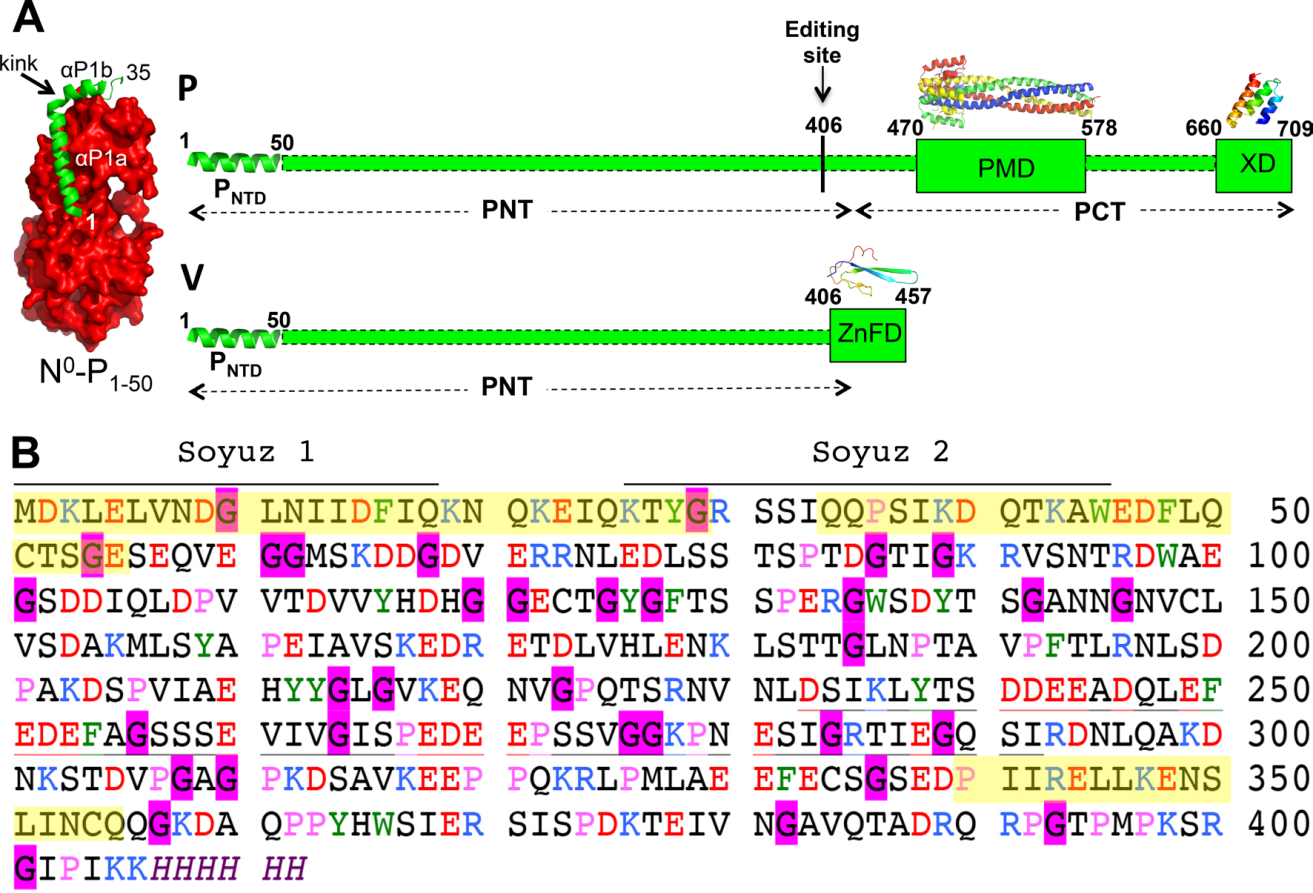
Modular organization of P and V, and amino acid sequence of NiV PNT. (**A**) Domain organization of P and V showing that P consists of two moieties, PNT and PCT, while V consists of PNT and of a zinc-finger domain (ZnFD). The P editing site is shown. Structured and disordered regions are represented as large and narrow boxes respectively. PNT: N-terminal region of P; PCT: C-terminal region of P. PMD: P multimerization domain; XD: X domain consisting of a triple α-helical bundle. The α-MoRE at the N-terminal region of P (P_NTD_), which is partly preconfigured in solution and shown to adopt a stable α-helical conformation upon binding to the monomeric form of N^4^, is shown as a green helix. The crystal structures of NiV PMD (PDB code 4N5B)^8^ and of the NiV N°-P_NTD_ complex (PDB code 4CO6)^4^ are shown. The homology-derived models of NiV XD^9^ and of the ZnFD of V are also shown^10^. All structures were drawn using Pymol 2.0.1 (https://pymol.org/2/)^11^. (**B**) Amino acid sequence of NiV PNT (Uniprot code Q9IK91). Basic and acidic residues are shown in blue and red, respectively. Aromatic residues are shown in green, prolines in pink and glycines in black on a pink background. The soyuz1 and soyuz2 motifs conserved in *Paramyxoviridae* members are shown. The low sequence complexity region (as obtained using SEG^12^ with a window size of 25 residues, trigger segment complexity of 3.0 and extension segment complexity of 3.3) is underlined. Fluctuating α-helices, as observed in NMR studies of NiV P sub-constructs^13^ are shown in yellow.

The repertoire of P functions is further expanded by the peculiar coding capacity of the P gene. Indeed, beyond the P protein, the P gene also codes for the V and W proteins that are generated through the addition of either one (protein V) or two (protein W) non-templated guanosines at the editing site of the P messenger. The addition of these guanosines triggers a downstream frame-shift. The P, V, and W proteins therefore share a common N-terminal region (referred to as PNT) that constitutes a *bona fide* domain (i.e. a genuine functional unit) as inferred from the genetic organization of the P gene (Fig. 1A). The *Henipavirus* V and W proteins are key players in the evasion of the interferon (IFN)-mediated response via an antagonist activity of IFN signaling^5,6^. V and W bind to STAT1, a key signal transducer in the IFN-mediated antiviral response, through their common PNT region^7^. Binding of STAT1 by V leads to inhibition of STAT1 translocation into the nucleus, whereas binding to W leads to sequestration of STAT1 in the nucleus^7^. The NiV P protein is endowed with anti-IFN function as well, indicating that the PNT domain common to P, V, and W is responsible for the IFN antagonist activity.

We previously showed that PNT from both NiV and HeV is intrinsically disordered^14^. The P C-terminal region (PCT) consists of an alternation of disordered regions and ordered regions (Fig. 1A). In fact, PCT comprises a disordered region (aa 407–469), referred to as « spacer » and overlapping with the reading frame of the C-terminal domain of V and W, a structured region responsible for the oligomerization of P (PMD, aa 470–578)^8,15^, a disordered linker and a structured region (X domain, XD, aa 660–709) with a triple α-helical bundle fold (Fig. 1A)^9^. We characterized the NiV and HeV V proteins and showed that PNT conserves its disorder also within the V protein, while the zinc-finger domain (ZnFD) has a predominant β conformation^10^ in agreement with predictions^16,17^. In that study, we also experimentally showed that the NiV and HeV V proteins interact with DDB1—a cellular protein whose binding to V promotes STAT1 degradation—and concluded that the ZnFD plays a crucial role in strengthening this interaction^10^.

Although our previous study on the V proteins from both NiV and HeV^10^ has contributed to illuminating the conformational behavior of these proteins and has provided a conceptual asset to design new antiviral strategies to combat the ability of these viruses to escape the innate immune response, a site-resolved description of these proteins is still lacking. A major hurdle in this respect arises from the presence of their intrinsically disordered PNT region, which prevents crystallization and whose size constitutes a challenge for NMR studies.

To fill this gap in knowledge, and as a first step towards an atomic description of the NiV V protein, we decided to investigate NiV PNT by combining small-angle X-ray scattering (SAXS) and NMR spectroscopy. When we started this project, high-resolution data were already available for the first 35 residues of NiV PNT. Yabukarski and co-workers indeed solved the X-ray structure of a complex (herein referred to as N^0^–P_1–50_) made of a truncated form of NiV N unable to self-assemble (aa 32–383) and of the first 50 residues of NiV P^4^ (Fig. 1A). Only the first 35 residues of P were defined in the electron density. This Molecular Recognition Element (MoRE) forms two α-helices (αP1a, residues 1–19; αP1b; aa 21–28) separated by a kink (Fig. 1A). In that study, the P region encompassing the first 100 residues (P_1–100_) was also investigated by NMR spectroscopy on a small construct. Although the HN correlation NMR spectrum is typical of a disordered protein, the secondary-structure propensities (SSPs) calculated from Cα and Cβ secondary chemical shifts indicate the presence of five fluctuating α-helices. Upon addition of the N protein, only residues 50–100 remain detectable indicating that they remain flexible in the complex and that the N^0^-binding region of P encompasses residues 1 to 50^4^. This latter region contains two conserved motifs in *Paramyxoviridae* members (i.e. *soyuz1* ad *soyuz2*)^18^ (see Fig. 1B).

When we were about to complete our NMR and SAXS characterization of NiV PNT, a study was published reporting the structural description of the NiV P protein^13^. In that study, the authors combined NMR spectroscopy, SAXS, and X-ray crystallography and obtained an ensemble description of this large protein. Notably, they decided to use a “*divide et impera*” approach to disentangle the NMR spectral complexity of the large disordered N-terminal region (residues 1–474). They thus generated several overlapping constructs that altogether cover the entire P protein and then assigned the HSQC spectrum of each construct corresponding to the P disordered regions and to XD. The chemical shift-derived SSPs of the fragments revealed that the long N-terminal intrinsically disordered region and the linker connecting PMD to XD dynamically sample multiple conformations while possessing short regions of residual secondary structure. The comparison between the HSQC spectrum of the full-length NiV P protein with the HSQC spectra of the individual P sub-constructs unveiled an overall good superimposition supporting a scenario where the N-terminal moiety remains highly flexible and retains a conformational behavior in the tetramer similar to that observed in the isolated sub-constructs, with negligible inter-chain contacts^13^.

Herein, we report the results we obtained using cutting-edge, multidimensional NMR spectroscopy approaches applied to the entire PNT domain. In spite of the challenging nature of such an assignment, we succeeded in assigning as much as 91% of the peaks of this long intrinsically disordered domain. The combination of SAXS and NMR information allowed us to obtain an ensemble description of the conformational behavior of the protein in solution.

## Results and discussion

### NMR residue-specific structural and dynamic characterization of NiV PNT

**Figure 2:**
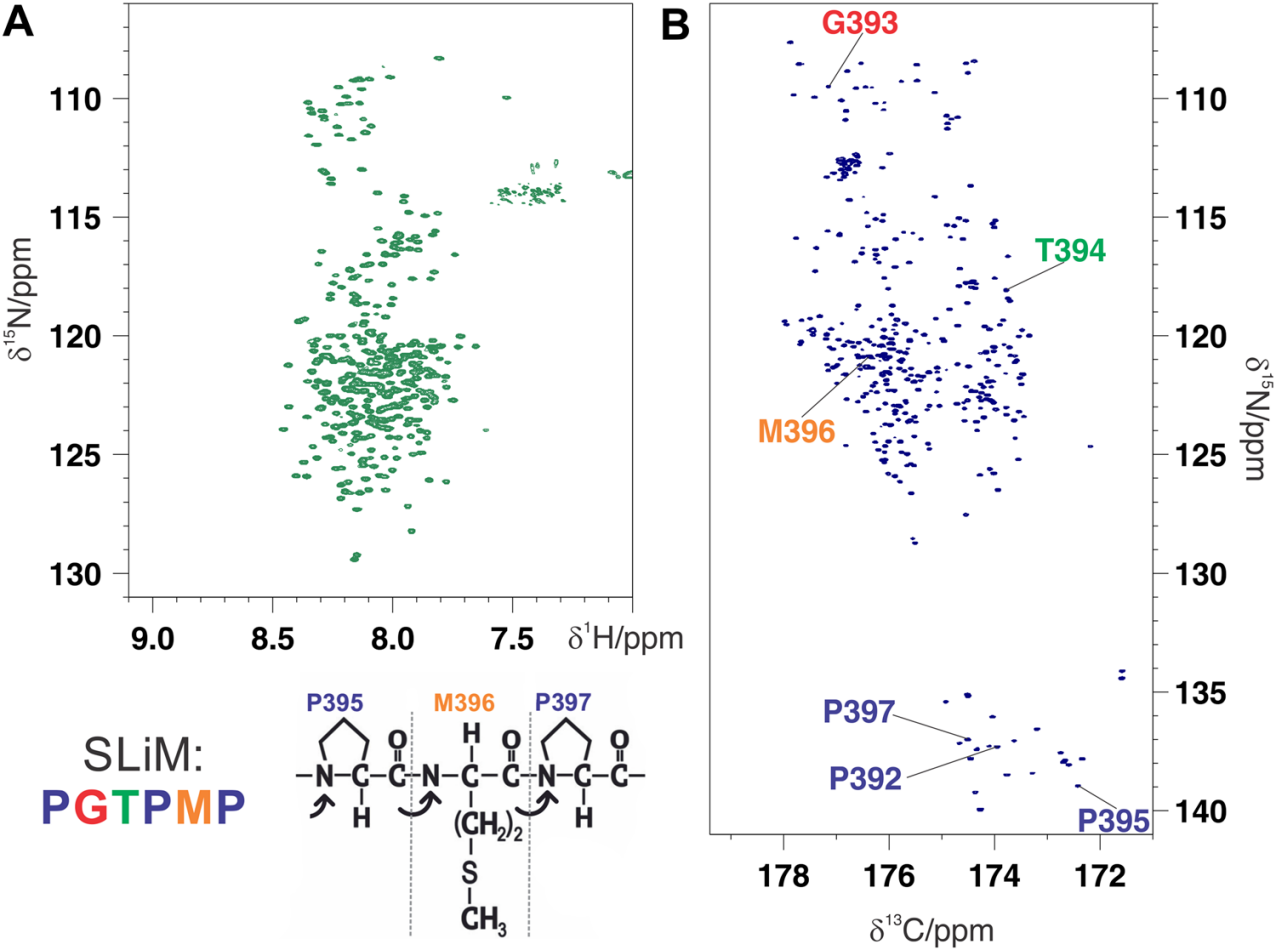
(**A**) 2D-^1^H^15^N-BEST-TROSY and (**B**) ^13^C-detected CON spectra  acquired on the full-length NiV PNT. The 2D CON spectrum is much more resolved and enables also the direct detection of the correlations involving the 26 prolines in a clean region of the spectrum. As an example of the importance of those residues, the cross peaks of the residues in a SLiM are reported. The complete assignment, including the ^13^C nuclei, is reported in Supplementary Table S2.

NiV PNT is a very large intrinsically disordered protein (412 residues including the C-terminal His tag and the initial methionine), as can be inferred by inspection of the two 2D spectra, the ^1^H–^15^N HSQC (Fig. 2A) and the ^13^C′–^15^N CON (Fig. 2B). The latter reveals also signals of proline residues and is characterized by improved chemical shift dispersion, two features that are very useful for the study of intrinsically disordered proteins (IDPs). An assignment strategy that combines ^13^C detected with ^1^H detected NMR is thus very helpful to provide sufficient information to enable sequence-specific assignment of the resonances of the full-length protein. A series of triple-resonance 3/5D NMR experiments, specifically designed for IDPs based either on ^1^H^N^ or ^13^C detection, were acquired^19,20^ (Supplementary Table S1). The projection reconstruction spectroscopy (APSY) approach was exploited in most of the multidimensional experiments to reduce the experimental time while preserving high spectral resolution in the indirect dimensions^21,22^.

The ^13^C′–^15^N CON spectrum of NiV PNT, shown in Fig. 2B, can be considered the reference spectrum for a series of multidimensional CON-based experiments in which correlations to additional nuclear spins in the indirect dimensions of the experiments provide the needed information to achieve the sequence-specific assignment. These multidimensional spectra include the ^13^C-detected 3D (H)CBCACON^23^, (H)CBCANCO^24^, (H)COCON^25,26^, and 5D (HCA)CONCACON^27^ spectra. Inspection of the spectra constituted the starting point for the sequence-specific assignment^22,27^.

This information was complemented with that available through ^1^H^N^ detected multidimensional NMR experiments. The 3D BEST-TROSY (BT) triple-resonance experiments^28^ HN(CO)CACB, HNCACB, HNCANNH, HNCO, HN(CO)CACB, HNCACB, and HNCOCANNH were acquired to obtain the complementary information needed to complete the assignment, augmented by a 5D BT-HN(COCAN)CONH experiment^29^ used to resolve a few ambiguities and to confirm the chemical shift assignment obtained with the 3D spectra.

**Figure 3:**
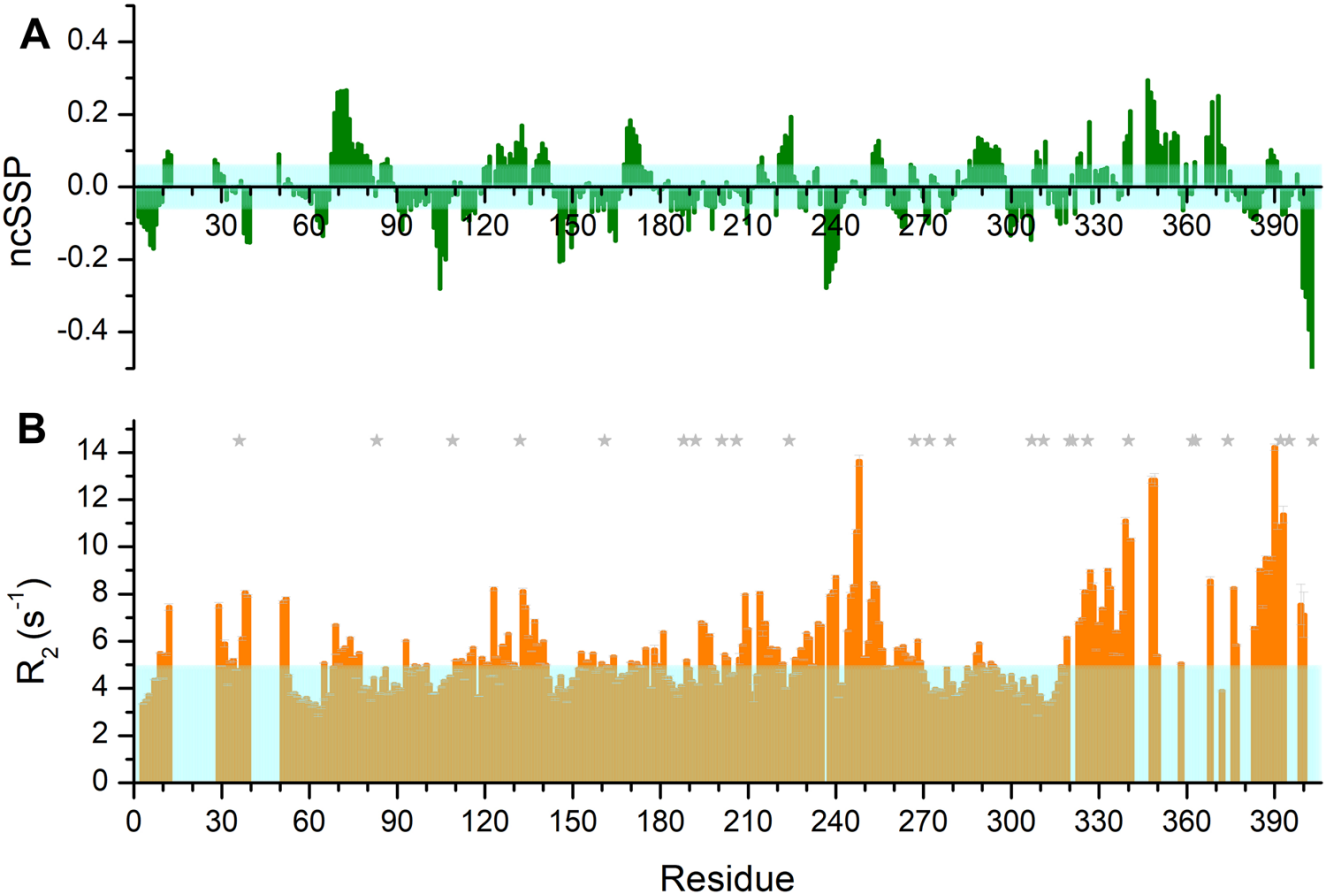
(**A**) Neighbor-corrected secondary structure propensity (ncSSP) values obtained through the online ncSSP tool (https://st-protein02.chem.au.dk/ncSPC/)^32^ using the chemical shift values reported in Supplementary Table S2. Positive and negative values correspond to α-helical and β-strand propensities, respectively. The light cyan box represents the ± 0.05 value. Few regions display a moderate α-helix propensity (SSP > 0.2) while most of the protein is largely disordered. (**B**) ^15^N R_2_ values reported as a function of residue number. The light cyan box indicates the modal value.

We could successfully assign the resonances of H^N^, N, C′, C^α^, and C^β^ nuclei of as many as 369 residues, including all proline residues (Fig. 2B), thus leading to a percentage of 91% of assigned residues for the entire protein. The chemical shifts of the previously mentioned nuclei are reported in Supplementary Table S2. The assignment is less complete in the N- and C-terminal moieties of the protein and in particular in the stretches encompassing residues 14–27, 41–49 and 342–346 either because of increased transverse relaxation or of residual signal overlap or both. The availability of heteronuclear chemical shifts enables the presence of residual secondary structure to be inferred^30^. The neighbor-corrected structural propensities (ncSSPs) were computed by comparing the experimentally measured chemical shifts of N, C′, C^α^, and C^β^ nuclei to the values expected for corresponding random coils^31^ using the tool available online at https://st-protein02.chem.au.dk/ncSPC^32^. The propensities to adopt α-helical and β-extended conformations, as obtained using the method of Mulder^32^, are shown in Fig. 3A and confirm that the protein is intrinsically disordered, with SSP values in the + /− 0.2 range for the majority of the primary sequence except for the regions encompassing residues 69–73, 237–240, 341–349, and 400–403.

Segments with SSP values between 0.05 and 0.2 indicate very modest deviations from random coil behavior. Moreover, the detection of local structural propensity in a polypeptide crucially depends on the choice of the reference chemical shifts for the random coil state and on possible bias induced by experimental conditions (e.g. buffer, pH, experimental temperature, etc.). Additional information is thus required to characterize the structural and dynamic properties of the protein in addition to nuclear chemical shifts.

To investigate backbone dynamics of NiV PNT, we measured heteronuclear ^1^H–^15^N NOE, ^15^N R_2,_ and ^15^N R_1_ values. We could successfully determine relaxation rates for most of the assigned cross-peaks observed in the 2D ^1^H–^15^N HSQC spectrum and discarded those whose overlap would have rendered the analysis ambiguous. The relaxation rates show an overall trend consistent with the considerably disordered conformation of PNT. ^1^H–^15^N NOE values are all well below 0.5, indicating high flexibility of the backbone (Supplementary Figure S1A). Contrary to the ^15^N R_1_ values that are rather uniform along the polypeptide chain (Supplementary Figure S1B), higher than modal ^15^N R_2_ values were observed in several regions of the primary sequence, i.e. 9–52, 110–141, 207–220, 226–256, 261–269 and 308–406 (Fig. 3B). The segments encompassing residues 9–52 and the C-terminal part of the polypeptide chain (340–406) are regions that are partially unassigned in our experimental conditions. This observation, as well as the absence of correlations in the multidimensional spectra, confirms that these regions are affected by dynamical phenomena leading to high R_2_ values, such as the presence of transiently populated secondary structural elements or chemical exchange broadening. The positive SSP values for the residues that can be observed in the regions 339–357 and 367–373 suggest the presence of transiently formed α-helices in these regions. The following stretch of about 30 amino acids, from residue 376 to the end of the protein primary sequence, is particularly rich in positively charged amino acids (22%), which suggests the possibility that the high nuclear relaxation rates observed in the absence of pronounced local secondary structural elements are due to the occurrence of transient electrostatic interactions (Fig. 1B and Supplementary Figure S2). Interestingly, the region encompassing residues 226–256 and 261–269, which overall shows higher than modal ^15^N R_2_, is rich in negatively charged residues (33%) and could indeed be engaged in transient interaction with the C-terminal, positively charged stretch (376–406).

It is also interesting to note that this latter region (i.e. 376–406) is rich in positively charged amino acids (with 7 of such residues), and also contains 4 prolines (Fig. 1B and Supplementary Figure S2). This particular amino acidic composition of alternating proline residues with amino acids sharing the same charge (positive in this case) could thus have a role in promoting conformations with a local net charge even in the absence of well-defined secondary structural elements. The same pattern of regions rich in amino acids sharing the same charge and proline residues is found in other regions, such as ^267^PEDEEP^272^, although in this case the amino acids are negatively charged. (Fig. 1B and Supplementary Figure S2). The NMR data thus suggest a possible cross-talk between regions enriched in residues with opposite charge. The 238–256 region is negatively charged and can establish interactions with positively charged regions, such as the C-terminal one encompassing residues 376–406 (Fig. 1B and Supplementary Figure S2).

Additional amino acid stretches showing higher than modal ^15^N R_2_ values are 110–141 and 207–220. These do not show evidence of transiently populated secondary structural elements nor highly charged segments. However, looking at the amino acid composition, in particular at amino acid types that are quite rare in IDPs^33^, one can observe a number of aromatic residues in these regions. These account for 22% of the residues in region 110–141 and 23% in region 207–220, indicating that these regions feature a higher amount of aromatic residues with respect to what is observed for the whole protein (6%) (Fig. 1B and Supplementary Figure S2). Aromatic residues are bulky amino acids and might be involved in local transient interactions without inducing specific secondary structural elements explaining the higher ^15^N transverse relaxation rates observed, as also observed for other IDPs^34^ in which aromatic residues have been shown to promote compact states.

Additional information that can be obtained by NMR derives from the investigation of exchange processes of amide protons with the solvent. Several CLEANEX experiments were acquired with different mixing times to measure the exchange rate constants^35^. Intensities of cross peaks are measured to obtain an estimation of the k_ex_ (s^−1^) (Supplementary Figure S1C). Thus, k_ex_ reports on solvent exchange processes of amide protons with the solvent on a per-residue basis. While this observable has been used extensively to characterize globular proteins and identify amide protons that are buried in hydrophobic cores and/or involved in hydrogen bonds through the determination of “protection factors”, in the case of IDPs it is not straightforward to interpret hydrogen exchange data in terms of structural effects as these are not easy to disentangle in a clear way from other effects such as local electrostatic potential, nature of the amino acid, and possible effects of neighboring amino acids in the primary sequence^36,37^. On the other hand, there is no doubt that this observable is rich in information^36–38^ and that, as we make progress in understanding the different contributions and in predicting exchange values for random coil conformations^39^, it might become a useful tool to achieve information about the structural and dynamic properties of IDPs.

Inspecting the observed k_ex_ for NiV PNT as a function of the primary sequence reveals that some regions present a k_ex_ that is higher than the modal value of 1.58 Hz, while some others are well below this value. Higher values are observed for residues 27–30, between two regions with large, positive SSP values at the edges of two transiently populated helices. Another region with high k_ex_ is the one encompassing residues 119–147, rich in aromatic residues (17%; Fig. 1B and Supplementary Figure S2), where slightly higher ^15^N R_2_ values were identified, in agreement with the presence of a solvent exposed region. Another region with significantly high rates is the one spanning residues 182–194, a region that is very rich in serine and threonine residues (38% in this segment *versus* 16% in the whole protein), two amino acids that are often solvent exposed and are characterized by high exchange rates with the solvent in general^39^ (Fig. 1B and Supplementary Figure S2). The region presenting lower k_ex_ values (slow chemical exchange) are the regions encompassing residues 97–122 and 239–263, which are rich in negatively charged residues (31% in the first segment, 40% in the second one) (Fig. 1B and Supplementary Figure S2), a feature that is known to cause reduced exchange processes^37,40^.

The NMR data clearly suggest that NiV PNT is disordered and very flexible with few short and transient secondary structure elements. The N-terminus shows moderate positive secondary structure propensity for residues 68–81, while some hints of the occurrence of other transiently populated α-helices can be obtained from the combination of ^15^N R_2_, SSP and k_ex_ values in the region encompassing residues 10–30. This region contains a segment (1–29) that adopts a helix-kink-helix conformation in the crystal structure of the N^0^–P complex^4^. Subsequent NMR studies confirmed the presence of two transiently populated α-helices at the N-terminus of NiV PNT also in solution and in the absence of the binding partner^4,13^. The presence of these helical elements mildly populated in our experimental conditions might be responsible for the conformational exchange processes likely causing the broadening of the signals in the full construct used here. Nevertheless, a few amino acids are detectable in this region and do show SSP values that indicate α-helical propensity (Fig. 3A). The more efficient transverse relaxation in these regions, as compared to the studies performed on shorter constructs, could be due to subtle differences arising from intramolecular interactions.

The overall behavior of PNT in solution might also be influenced by the experimental conditions. Indeed, the buffer used in^13^ includes compounds that mitigate protein self-association and aggregation (150 mM NaCl, 50 mM arginine, 50 mM glutamate)^41^. It should be pointed out however that in other cases the same buffer was reported to induce protein compaction by minimizing the effect of intramolecular long-range electrostatic interactions^42^. The causes of these conflicting effects are not well understood^42^.

Similar considerations also hold for the 340–355 region that was shown by Jensen and co-workers to partly sample an α-helical conformation^13^. Although we could not map the core region of the transient α-helix we mapped the flanking residues that, also in this case, confirm the presence of a transient α-helix in this region. This transiently populated α-helix may correspond to a binding site for one or multiple partners whose identification will require future studies. A possible partner could be the unassembled form of the N protein, by analogy with measles virus. Indeed, the counterpart of this region in measles virus P corresponds to a transiently populated α–helix (α4, aa 190–198) that binds weakly N^0^^43^.

The transiently populated α-helices are in good agreement with predictions as obtained with both FESS (the fast secondary structure predictor implemented in FELLS^44^ (Supplementary Figure S2) and PSIPRED^45^ (Supplementary Figure S3A). In terms of predicted MoREs (see Supplementary Figure S3A), a quite good agreement was found with the experimental data, as well as with already mapped binding sites, including the STAT1 (aa 110–140)^13^ and STAT2 (aa 110–140 and 230–237) binding sites^46^. The 110–140 region shown to constitute a weak binding site to STAT1^13^ does not exhibit significant SSP values (all the values are below 0.2), a finding that suggests that this region might correspond to an I-MoRE (Irregular-MoRE), i.e. a region that may remain at least partially disordered after binding to the partner. This latter hypothesis is further corroborated by the fact that addition of STAT1 only triggers a decrease in the resonance intensities of the 110–140 region with no concomitant peak shift^13^.

An analysis performed on the ELM database (https://elm.eu.org/)^47^ highlighted the presence of numerous Short Linear Motifs (SLiMs) along the sequence. SLiMs, also known as linear motifs (LMs), are short stretches of adjacent amino acids mediating protein–protein interactions and occurring within IDRs^48^. SLiMs play crucial roles in cell regulation and SLiM mimicry is often used by viruses to hijack their host cellular machinery^49^. A nice correlation was found between the presence of SLiMs and the dynamic behavior of NiV PNT. Indeed, 22 SLiMs were identified (Supplementary Table S3) and most of them (18) are located within the regions displaying high R_2_ values (Supplementary Figure S3B). As an example, the previously mentioned C-terminal region (aa 376–406), enriched in proline residues and in positively charged residues, features several (i.e. 7) SLiMs. These SLiMs are mainly involved in interactions with kinases, phosphatases, SH3 and WW regulatory domains, consistent with a role in hijacking key cell regulatory processes. These interactions can now be investigated at atomic resolution through ^13^C-detected experiments tailored for proline residues that allow acquiring quick snapshots in a clean spectral region^50,51^.

### SAXS ensemble characterization of NiV PNT

**Figure 4:**
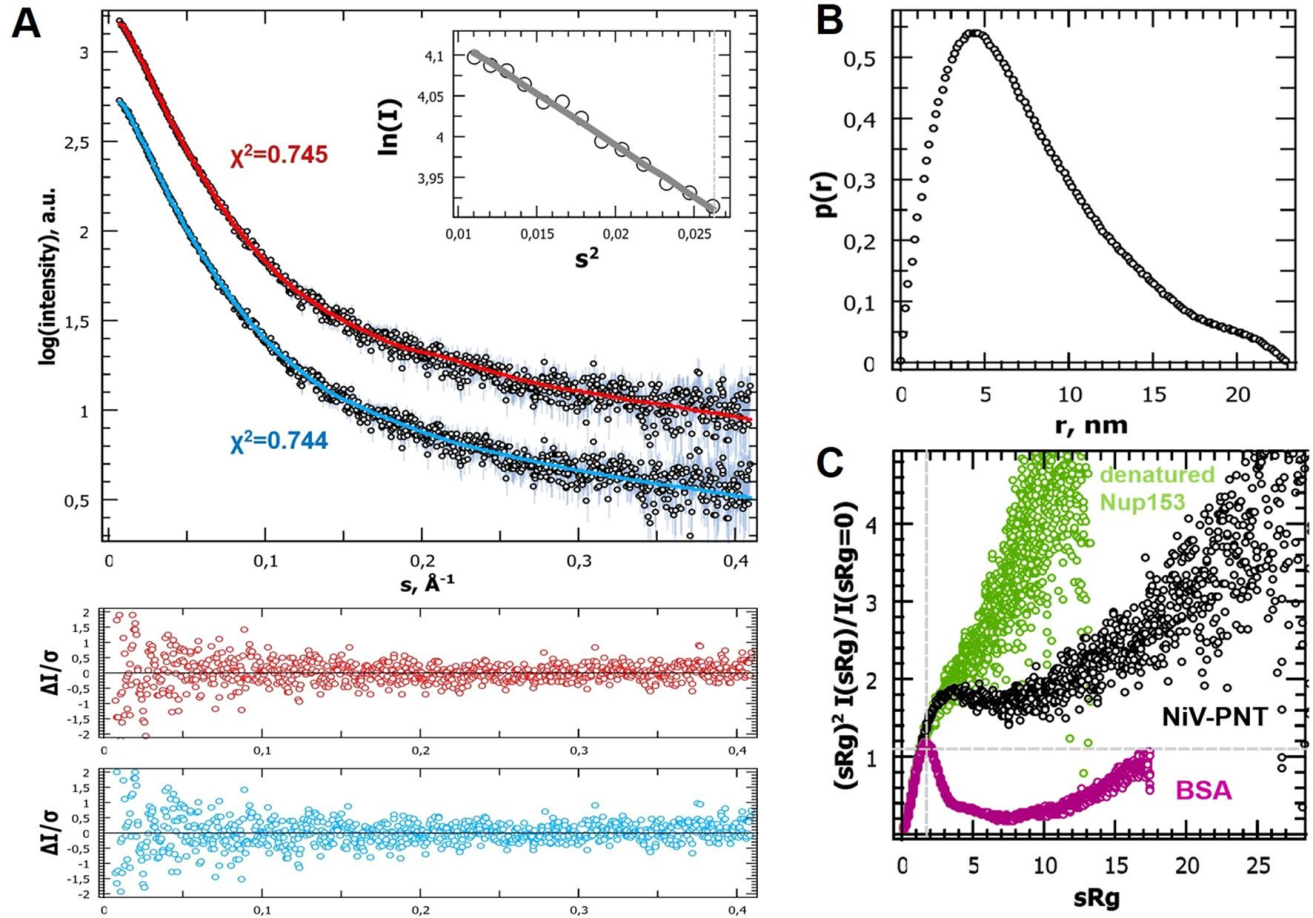
SAXS studies of NiV PNT. (**A**) SAXS scattering curve of NiV PNT and ensemble EOM 2.0 fits extracted from two distinct initial pools: (*blue*) generated by Flexible-Meccano considering ncSSP and (*red*) generated by EOM 2.0 including random Cα-only conformers. Inset: Guinier plot of the experimental scattering curve. (**B**) Pair distance distribution function, P(r). (**C**) Normalized Kratky plot representation of the scattering data. The normalized scattering plots of a globular (BSA, SASDA32) and denatured (Nup153, SASDEY2) protein are shown for comparative purposes to illustrate that NiV PNT has an overall conformation in between these two extreme examples.

Synchrotron SAXS in solution measurements were performed to gain insight into overall NiV PNT conformation and motion. Linearity in the *Guinier* region at low angles (*sR*_*g*_ < 1.0) revealed good data quality, with no indication of protein aggregation (Fig. 4A, inset). The molecular mass determined from the forward scattering intensity at zero angle I(0) was ~ 47 kDa indicating a monomer in solution and in agreement with the value (45.3 kDa) computed from the amino acid sequence using the expasy server (https://web.expasy.org/protparam/). Although not significantly different, the *R*_*g*_ extracted from *P*(*r*) is slightly larger (~ 63 Å) than the one extracted from *Guinier* analysis (~ 61 Å). The theoretical *R*_*g*_ value expected for an IDP of the same length (412 amino acids) calculated using Flory’s power law ${R}_{g}^{IDP}={R}_{0}{N}^{v}$, where R_0_ is 2.54 ± 0.01, N is number of amino acid and $v$ is 0.522 ± 0.001^52^, is ~ 59 Å. The experimentally determined R_g_ is therefore very close to the value expected for an IDP (for comparison the R_g_ expected for a random coil conformation would be ~ 72^53^).

*P*(*r*) yields a maximal dimension, D_max_, of ~ 230 Å (Fig. 4B) with a long tail in the *P*(*r*) function, suggesting that the protein tends to assume an overall non-compact conformation^54^. The overall SAXS parameters for NiV PNT are listed in Supplementary Table S4.

The flexible nature of NiV PNT was qualitatively assessed by using the normalized Kratky plot, where the absence of a well-defined bell shape indicates a protein with intrinsically disordered regions (Fig. 4C). To achieve further insights into the conformational behavior of NiV PNT, data obtained from NMR and SAXS experiments were combined in search of an ensemble that quantitatively describes the conformational behavior of NiV PNT in solution. In a first step, a pool of 50,000 structures was generated based on the NMR data. To this end, the ncSSP values larger than ± 0.05 were provided as inputs to Flexible-Meccano^55^. Subsequently, from the NMR-based pool, we used Ensemble Optimization Method (EOM) 2.0 to generate a conformational sub-ensemble that best fits the experimental SAXS data. In order to minimize over-fitting, EOM attempts at minimizing the number of conformers able to fit the experimental data and usually generates ensembles consisting of 5 to 40 conformers. Based on the high flexibility expected, no repetition of conformations in the ensemble was allowed. The scattering curve back-calculated from the selected ensemble (Fig. 4A, *cyan curve*) fits well the experimental SAXS data as judged from both *χ*^2^ and CorMap^56^ (*χ*^2^ = 0.744, *P* = 0.101). The resulting final *R*_*g*_ distribution, broader than the one generated from the NMR-based pool, indicates that NiV PNT exists in solution as a randomly distributed ensemble of non-compact and highly flexible conformations. Note that successive and independent selections by EOM 2.0 consistently yielded similar R_g_ distributions thus attesting the reproducibility of the results (data not shown). The flexibility of the ensemble was quantified as Ensemble_Rflex_ =  ~ 91% (*NMR-Pool*_*Rflex*_ =  ~ 83%).

The average R_g_ value of the ensemble (66 Å) slightly exceeds the theoretical value expected from Flory’s power law (59 Å). This discrepancy may reflect sequence specificities, i.e. specific sequence attributes such as proline content and charge decoration (i.e. net charge per residue, fraction of charged residues and linear distribution of opposite charges) that were shown to be major determinants of IDP conformational properties^57–62^. For an additional discussion of the observed discrepancy see Supplementary text.

**Figure 5:**
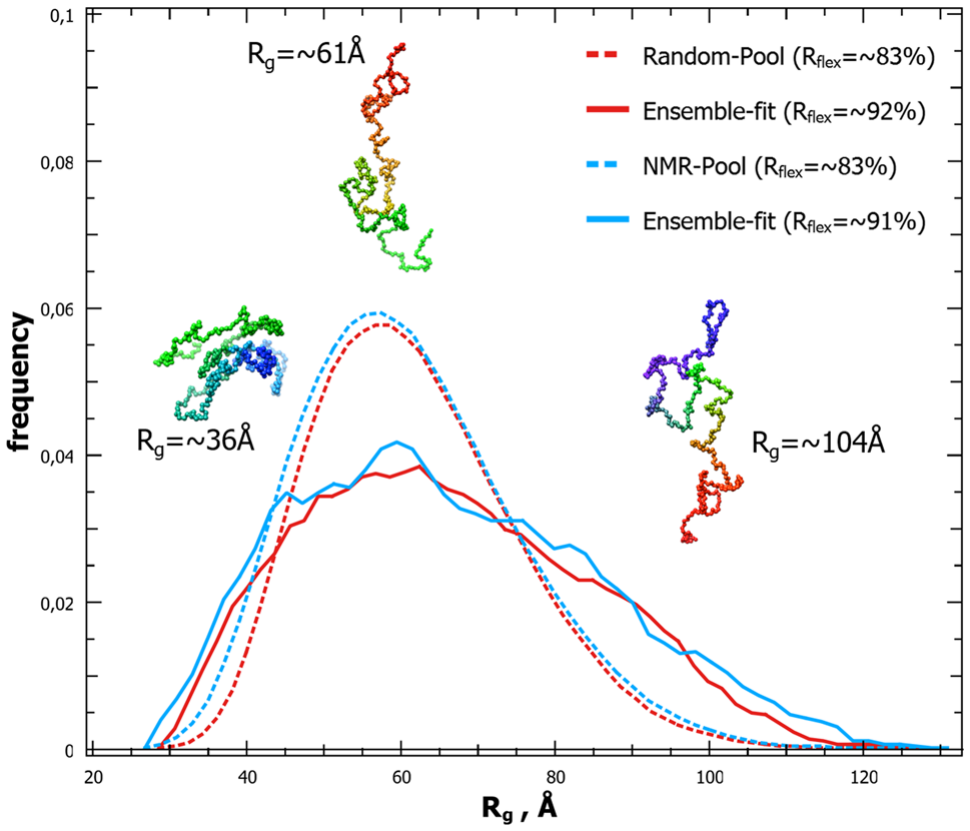
Modeling NiV PNT as a conformational ensemble. *R*_*g*_ distributions of the initial pools generated by (*blue dot*) Flexible-Meccano (with NMR secondary structure restraints, $\overline{{R_{g} }}$ =  ~ 62 Å) and (*red dot*) EOM 2.0 (without restraints, $\overline{{R_{g} }}$ =  ~ 62 Å) respectively, and of the corresponding selected ensembles (*solid lines*) as obtained using EOM 2.0 (both $\overline{{R_{g} }}$ =  ~ 66 Å, see also Fig. 4A). Final best ensembles contain 19 unique conformers in the case of ncSSP NMR-based ensemble and 13 unique conformers in the case of EOM 2.0 random ensemble, respectively. A cartoon representation of three conformers present in the ensembles is also displayed. The structures were drawn using Pymol 2.0.1 (https://pymol.org/2/)^11^.

A similar investigation was conducted by using a random pool generated by EOM 2.0 without NMR-based restraints (*χ*^*2*^ = 0.745, *P* = 0.192) (Fig. 4A, *red curve*). EOM 2.0 yielded similar results with randomness quantified as *Ensemble*_*Rflex*_ =  ~ 92% (*Random-Pool*_*Rflex*_ =  ~ 83%) such that no significant differences was observed when using different pools (Fig. 5).

Although SAXS is poorly sensitive to secondary structure and although the presence of secondary structure elements has been reported to have a limited impact on protein compaction^57,63^, we sought at investigating the impact of the occurrence of the N-terminal transiently populated α-helix on chain compaction. To this end, using Flexible-Meccano, we generated an initial pool in which an α-helical conformation was imposed to residues 1–30, with the experimental ncSSPs. Furthermore, we also investigated the final *R*_*g*_ distribution while imposing a kink centered at residue 20 (as observed in the crystal structure of the N^0^–P_1–50_ complex) providing NMR restraints. All the resulting sub-ensembles fit equally well the scattering data as judged from the obtained χ^2^ values and even for these two cases, no differences were observed in terms of the resulting *R*_*g*_ distributions (Supplementary Figure S4). This probably reflects the fact that SAXS is a low-resolution technique that provides relevant information in terms of chain compactness but fails to capture secondary structure propensities. In addition, previous findings from others and us have highlighted that regular secondary structure does not overly contribute to protein compaction (i.e. more compact forms do not necessarily exhibit an increased content in regular secondary structure)^57,63^. As such, it is not surprising that the presence of transiently populated α-helices in the initial ensemble does not have a significant impact on the final SAXS-derived sub-ensembles. Nevertheless, comparing SAXS-only ensembles to ensembles generated by the combined use of NMR and SAXS restraints is of interest given the growing interest towards ensemble descriptions of IDPs. In addition, combining NMR and SAXS data enables at least partly overcoming over-fitting as already discussed by Mertens and Svergun^64^.

## Conclusions

Albeit IDPs/IDRs are notoriously prone to undergo proteolytic degradation, we managed in generating a full-length NiV PNT sample stable enough to allow acquiring all the NMR spectra required for the assignment. Although the length of this intrinsically disordered domain is very challenging for NMR studies, the use of multidimensional NMR spectroscopy and of ^13^C-detected experiments allowed us to assign as much as 91% of the residues including prolines. The combination of SAXS and NMR data led to ensemble models of NiV PNT showing its conformational heterogeneity.

The presence of such a large disordered region shared by both the V and P proteins is likely related to their promiscuity. Structural disorder is known to serve as a determinant of protein interactivity^65–67^. Indeed V interacts with various cellular partners, such as DDB1^10^, STAT proteins^13,46,68^, CRM1^46,69^, PLK1^70^ and, possibly, nuclear factors such as IRF3. All these interactions play a crucial role in the ability of the virus to counteract the antiviral innate immune response of the host. The P protein binds to multiple partners as well, including N and the L protein (for a review see^71^). Therefore, intrinsic disorder represents an ergonomic solution for the virus to encode fewer proteins with more functions while keeping the genome size as small as possible. It is therefore not surprising to find large IDRs in proteins having a broad molecular partnership, such as the *Henipavirus* V and P proteins, and, more generally, in viral proteins^72,73^.

Intrinsic disorder also represents a strategy to alleviate constraints imposed by evolution on overlapping reading frames (such in the case of PNT that overlaps with the C protein) and to buffer the deleterious effect of mutations (i.e. IDRs/IDPs are more tolerant of substitutions compared to globular proteins) (for reviews see^71,73^). It is also well established that intrinsic disorder allows affinity and specificity to be uncoupled (i.e. it enables interactions with low affinity and decent specificity) (see^33,74,75^ and references therein cited). However, the occurrence of residual disorder in complexes involving IDPs (in the form of fuzzy appendages) and partial preconfiguration of binding motifs before binding afford a way to attenuate the entropic penalty associated to the disorder-to-order transition thereby, ultimately, modulating the binding affinity. The involvement of IDRs in protein–protein interactions that need to be finely tuned offers an exquisite means to modulate the strength of those interactions: by tuning the extent of preconfiguration of the binding motifs and/or the length of flanking fuzzy appendages, the virus can reach an optimal binding strength. It is therefore conceivable that the presence within NiV PNT of long disordered regions flanking binding motifs, including SLiMs, as well as binding sites to N^0^ or STAT proteins, may exert a role in regulating these critical interactions. The flexibility of these flanking regions would enable regulating both the exposure of binding sites specifically recognized by multiple viral and cellular partners, and the affinity of these interactions. Ultimately, this would result in their ability to orchestrate virus replication, through hijacking of cellular pathways and evasion of the IFN response.

The present results constitute an asset for obtaining atomistic information in future interaction studies involving NiV PNT. Indeed, the availability of the chemical shifts of full-length NiV PNT will be instrumental to map the residues involved in binding to partners such as DDB1 and/or STAT1 in the context of either the isolated PNT domain or the V protein. Once detailed information is available from interaction studies, inhibitors can be rationally conceived. Therefore the present study can be regarded as a starting point towards the design of inhibitors abrogating the ability of this virus to escape the innate immune response. Given the high similarity between NiV and HeV PNT (56%), it is conceivable that the results of these studies could be extrapolated to some extent to the HeV as well.

## Methods

### Protein expression and purification

The NiV PNT construct, encoding residues 1–406 of the NiV P protein with a C-terminal hexahistidine tag, has been already described^14^. Expression of unlabeled NiV PNT was carried out as previously described^14^.

Isotopically labeled (either ^15^N–^13^C or ^15^N) NiV PNT samples were prepared by growing at 37 °C transformed *E. coli* T7 cells (New England Biolabs, Ipswich, MA, USA) bearing the pRARE plasmid (Novagen, Madison, WI, USA) in LB medium supplemented with 100 μg mL^−1^ ampicillin and 34 μg mL^−1^ choramphenicol. When the OD_600_ reached 0.6, the culture was centrifuged at 4000 rpm for 10 min and the pellet was resuspended in ¼ of the initial volume of M9 medium (6 g L^−1^ of Na_2_HPO_4_, 3 g L^−1^ of KH_2_PO_4_, 0.5 g L^−1^ of NaCl, 0.246 g L^−1^ of MgSO_4_) supplemented with 1 g L^−1^ of ^15^NH_4_Cl and 2 g L^−1^ of either glucose or ^13^C-glucose. After one hour at 37 °C, IPTG was added to a final concentration of 0.5 mM, and the cells were subsequently grown at 37 °C for 3.5 h.

NiV PNT was purified as described in^14^, except that 6 M GuHCl was added after the lysis step to both denature bacterial proteases and recover the recombinant protein also from inclusion bodies with the aim of improving protein stability and yield. After 1 h incubation at 4 °C, the sample was clarified and the supernatant was purified through immobilized metal affinity chromatography (IMAC). The fractions containing the recombinant protein were combined, and then loaded onto a Superdex 75 h 16/60 column (GE, Healthcare). The elution buffer was 10 mM sodium phosphate pH 6.5, supplemented with 5 mM EDTA and 5 mM DTT. The fractions containing the protein were collected and conserved at − 20 °C.

### NMR spectroscopy experiments

Immediately before NMR studies, the NiV PNT sample was thawed, centrifuged at 12,000 rpm to remove any possible protein aggregate and then concentrated using 30 kDa Amicon Ultra Centrifugal Filters (Merk Milllipore, Darmstadt, Germany). All NMR spectra for assignment were acquired on ^13^C–^15^N-isotopically enriched NiV PNT at a concentration of about 200 µM.

The ^1^H-detected spectra were acquired at 288 K with a 22.3 T Bruker Avance III 950 NMR spectrometer; the ^13^C-detected spectra were acquired at 288 K with a 16.4 T Bruker Avance NEO 700 NMR spectrometer equipped with a cryogenically cooled probehead optimized for ^13^C-direct detection. Experimental details are reported in Supplementary Table S1.

All the heteronuclear relaxation experiments (R_1_, R_2_ and ^1^H–^15^N NOEs) were acquired using ^15^N labeled NiV PNT at about 100 µM. The spectra were recorded at 288 K on a 16.4 T Bruker Avance NEO 700 NMR spectrometer equipped with a cryogenically cooled triple resonance probehead. The ^15^N R_1_ and R_2_ experiments were acquired with 8 scans (2048 × 256 points) and a relaxation delay of 3.0 s. To determine the ^15^N R_1_ the following delays were used: 20 ms, 60 ms, 120 ms, 180 ms, 250 ms, 400 ms, 500 ms, 600 ms, 750 ms, and 900 ms. To determine the ^15^N R_2_ the following delays were used: 32 ms, 64 ms, 96 ms, 128 ms, 160 ms, 190 ms, 260 ms, 320 ms, 380 ms, 440 ms, and 500 ms. The ^1^H–^15^N NOEs experiments were acquired with 64 scans (2048 × 288 points) and a relaxation delay of 6.0 s.

The CLEANEX experiments were acquired on a 100 µM sample, with 16 scans (2048 × 248 points) and a relaxation delay of 3.0 s. The following delays were used: 5 ms, 10 ms, 20 ms, and 30 ms.

### NMR data processing and analysis

NMR data sets were processed using the Bruker TopSpin 4.0.6 software. CARA^76^ and its tool XEASY^77^ were used to analyze and annotate the spectra.

The ^15^N relaxation rates (R_1_ and R_2_) were determined by fitting the cross-peak intensity measured as a function of variable delay, to single-exponential decay using the Bruker Dynamic Center 2.4, available as a stand-alone ancillary software of TopSpin by Bruker. ^1^H–^15^N NOE values were obtained as the ratio between peak intensity in spectra recorded with and without ^1^H saturation.

The secondary structure propensity (SSP) from heteronuclear chemical shifts was determined by using the neighbor corrected structural propensity calculator (ncSPC) tool^32^ available online at https://st-protein02.chem.au.dk/ncSPC. The Mulder random coil chemical shift library^31^ was chosen for the analysis, and the average window size was left to the standard value of 5.

### SAXS experiments and analysis

Samples were concentrated using 30 kDa Amicon Ultra Centrifugal Filters (Merk Milllipore, Darmstadt, Germany) and loaded onto a Superdex 75 h 16/60 column using 20 mM Tris/HCl pH 8.0, 0.3 M NaCl, 5 mM DTT as elution buffer. Synchrotron X-ray scattering data were collected at ESRF BM29 beamline (Grenoble) using a PILATUS 1 M pixel detector (DECTRIS, Baden, Switzerland) at a sample-detector distance of 2.87 m and a wavelength of 0.0992 nm. This setup covers a range of momentum transfer of 0.028 < *s* < 4.525 nm^–1^ (*s* = *4π sin*(*θ*)*/λ*, where *2θ* is the scattering angle). Samples were loaded using a robotic sample changer^78^ and measured at 20 °C at concentrations ranging from 0.5 to 1.2 mg/mL. 10 independent frames of 1 s each were collected for each sample and data were automatically reduced using an in-house pipeline. Further analysis was done using ATSAS 3.0.1^79^. As no concentration dependence was observed (Supplementary Figure S5), the curve with less noise (at 1.2 mg/mL) was used for the SAXS analysis. The forward scattering *I*(0) as well as the *R*_g_ were calculated using the Guinier approximation assuming that, at very small angles (*s* < 1.3/*R*_g_), the intensity is represented as *I*(*s*) = *I*(*0*) · *exp*(*− *(*sR*_g_)^2^*/*3)^80,81^. The forward scattering intensities were calibrated using water as reference. Linearity in the Guinier region was used to exclude sample aggregation, and the pair-distance distribution function, *P*(*r*), from which the *D*_max_ and the *R*_g_ were estimated, was computed using GNOM^82^. Qualitative assessment of compactness *versus* structural disorder was made by transforming the scattering profiles in the so-called normalised Kratky representation [(*sR*_g_)^2^ * I(*s*)*/I*(*0*) vs. *sR*_g_]^83^. The MM was derived by placing the scattered intensity on an absolute scale using liquid water as calibrant.

Protein flexibility was quantified using EOM 2.0^84^ that assumes coexistence of a range of Cα-only conformations in solution for which an average scattering intensity fits the experimental data. A genetic algorithm (GA) is used to select ensembles with varying numbers of conformers. The GA is repeated *n* independent times, and the ensemble with the lowest discrepancy considered as the best solution out of *n* final ensembles. Furthermore, repetition of GA allows the computation of *R*_g_ distributions so that structural information about the flexibility could be extracted. The width of the distribution is used to derive the flexibility of the particles, whereby a narrow distribution indicates a rather rigid particle and broader distributions are associated with higher flexibility. Using EOM 2.0, systematic quantification of the flexibility was made by using the metric *R*_flex_—which computes the Shannon information entropy of the distributions^84^. Experimental error-independent goodness-of-fit was also confirmed by using the software CorMap that estimates the differences between one-dimensional spectra independently of explicit error estimates, using only data point correlations^56^. All the softwares used for the SAXS data analysis are part of the ATSAS 3.0 package^79^.

### Accession numbers

The chemical shifts have been deposited in the Biological Magnetic Resonance Data Bank (BMRB) under the code 50370. SAXS data have been deposited in the Small Angle Scattering Biological Data Bank (SASBDB)^85^ under code SASDJB5. The ensemble derived using SAXS constraints only and the one derived from the combined use of SAXS and NMR constraints have been deposited within the Protein Ensemble Database (PED-DB, https://proteinensemble.org/)^86^ under accession numbers PED00176 and PED00177, respectively. All-atoms pdb files, required for deposition in the PED-DB, were generated from EOM Cα-only pdb files using the PD2ca2main server (http://www.sbg.bio.ic.ac.uk/~phyre2/PD2_ca2main/)^87^.

## Supplementary information

Supplementary Information.
