# Supplementary material for: Ensemble description of the intrinsically disordered N-terminal domain of the Nipah virus P/V protein from combined NMR and SAXS
Source: Sci Rep. 2020 Nov 11;10:19574. doi: 10.1038/s41598-020-76522-3 (PMC7658984; doi:10.1038/s41598-020-76522-3)
Supplement: Supplementary file 1 — Supplementary Information. [file 41598_2020_76522_MOESM1_ESM.pdf]

Supplementary Material

Ensemble description of the intrinsically disordered N-terminal domain of the Nipah virus P/V protein from combined NMR and SAXS

Marco Schiavina[1,2$], Edoardo Salladini[4$], Maria Grazia Murrali[1,2$], Giancarlo Tria[2,3], Isabella Felli[1,2]*, Roberta Pierattelli[1,2]* and Sonia Longhi[4]*

[1]Magnetic Resonance Center (CERM), University of Florence, Via Luigi Sacconi 6, 50019 Sesto Fiorentino, Italy
[2]Department of Chemistry "Ugo Schiff", University of Florence, Via della Lastruccia 3-13, 50019 Sesto Fiorentino, Italy
[3]Florence Center for Electron Nanoscopy (FloCEN), University of Florence, Via della Lastruccia 3-13, 50019 Sesto Fiorentino, Italy
[4]Aix Marseille Univ, CNRS, Architecture et Fonction des Macromolécules Biologiques (AFMB), UMR 7257, Marseille, France

*To whom correspondence should be sent:

felli@cerm.unifi.it
roberta.pierattelli@unifi.it
sonia.longhi@afmb.univ-mrs.fr; sonia.longhi@univ-amu.fr

[$]These authors contributed equally to the work

Supplementary text

Discrepancy between experimentally observed R_g and R_g expected from Flory's power law

Flory's power law attempts at generalizing the IDP behavior while taking into account only sequence length and thus neglecting sequence specificities. Although Flory's power law remains a useful tool, the extent to which it can be generalized to all IDPs is a matter of debate, with several instances of deviations having been experimentally observed. Systematic collections of SAXS data on IDPs, such as the work of Cordeiro et al. [1], show a large scatter that indicates a lot of variation in scaling behavior. A similar study carried out Sosnick and co-workers [2] showed that several IDPs obeyed a scaling law with an exponent close to 0.6. Further, Sosnick and co-workers explained that the scaling law exponent reflects the quality of the solvent (aqueous buffer, sometimes also with denaturant) for a particular IDP and that solvent quality (and scaling exponent) can vary with the amino acid sequence. Consequently, it is not surprising to observe deviations from Flory's power law such as those we observed for NiV PNT.

Supplementary Figures

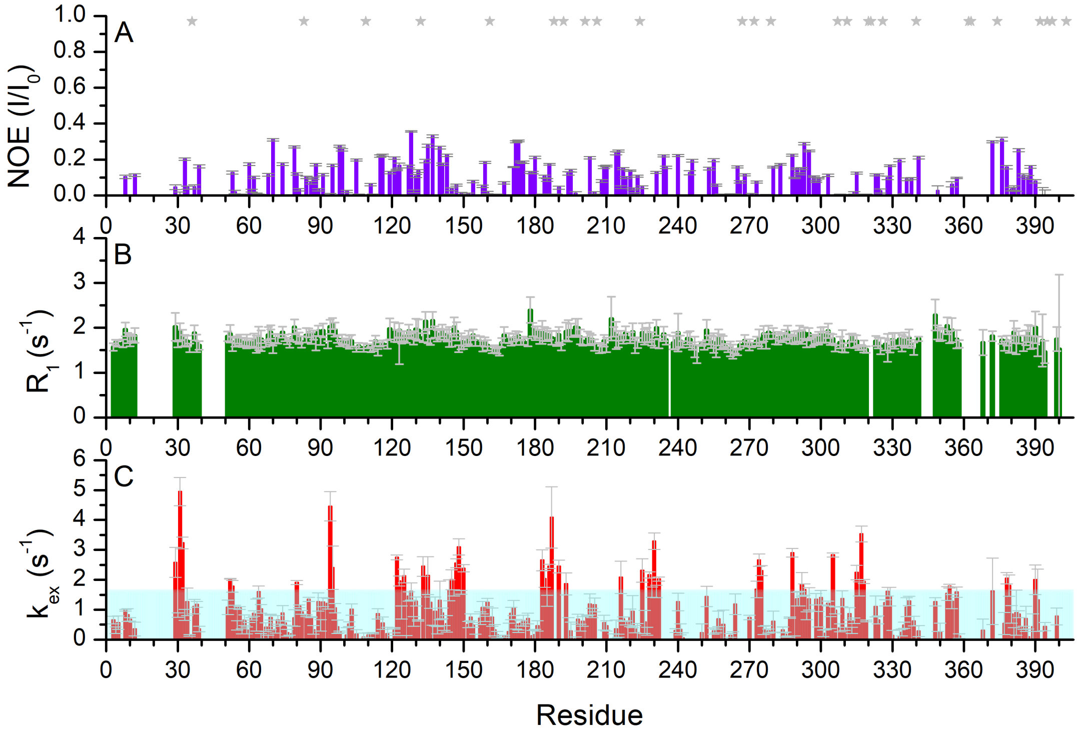

Supplementary Figure S1. (A) ^{1}H-^{15}N-NOE measurements, **(B)** ^{15}N R1 relaxation rates and **(C)** k_{ex}, as derived from CLEANEX-PM experiment using the approach reported in [3], of NiV PNT, carried out at 700 MHz and 288 K on a 100 μM ^{15}N-labeled sample. The light cyan box represents the modal value of 1.38 s^{-1}.

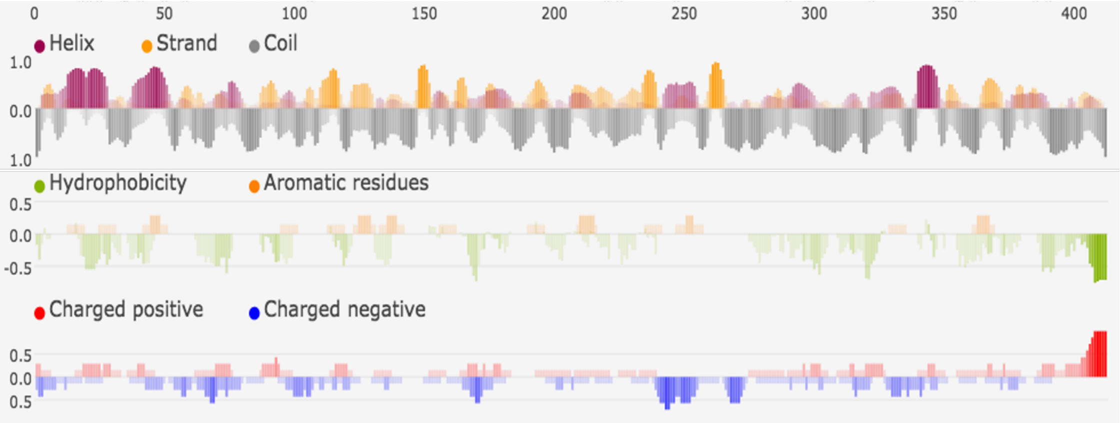

Supplementary Figure S2. Excerpt of the output generated by FELLS [4].

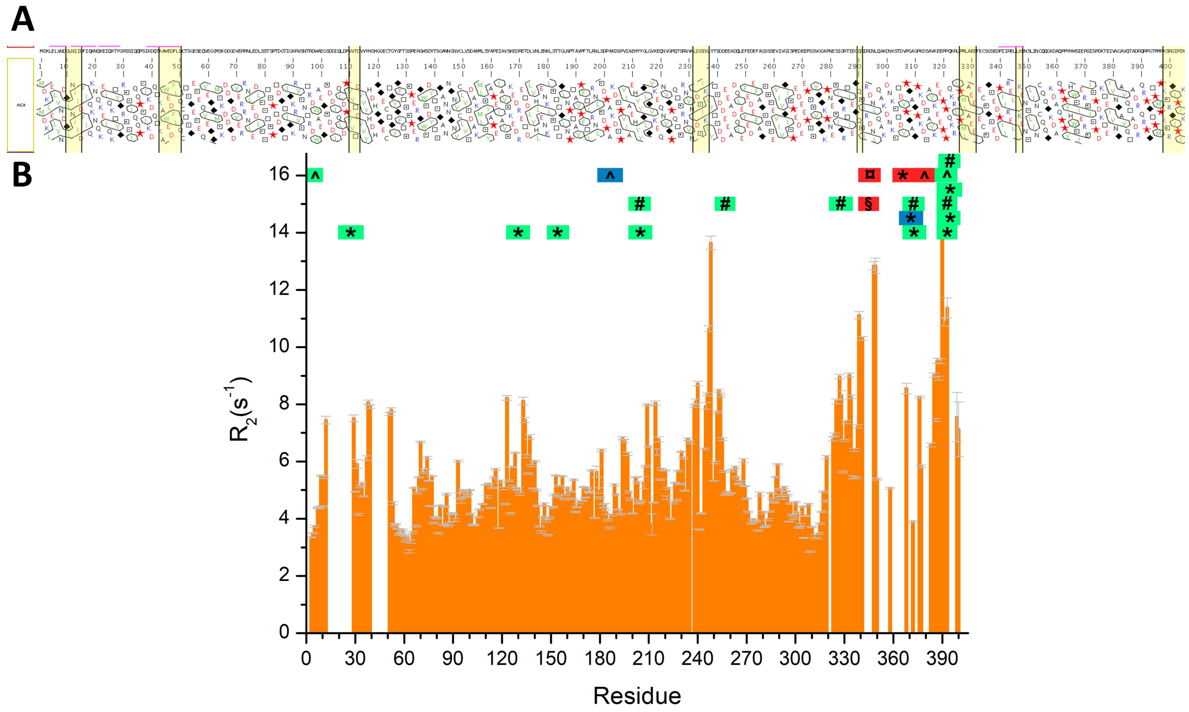

Supplementary Figure S3. (**A**) Hydrophobic cluster analysis (HCA) plot [5] of NiV PNT as obtained using MeDor [6]. Molecular Recognitions Elements (MoREs), as predicted by MoRFPred [7] are shown in yellow. The α-helices, as predicted by PSIPRED [8], are shown as pink horizontal bars above the sequence. Four of them fall within the region encompassing the first 50 residues while the fifth one encompasses residues 340-348. (**B**) ^{15}N R_2 relaxation rates and putative Short Linear Motifs (SLiMs) as identified by the ELM database (http://elm.eu.org/) (see also **Supplementary Table S3**). The SLiMs are identified as follows according to the ELM classification: §, CLV (cleavage site); #, DOC (docking site); ^; LIG (ligand binding site); *, MOD (post-translational modification site), ¤, TRG (targeting site). The color code is representative of the conservation score (red < 0.95; blue 0.95-1; green 1).

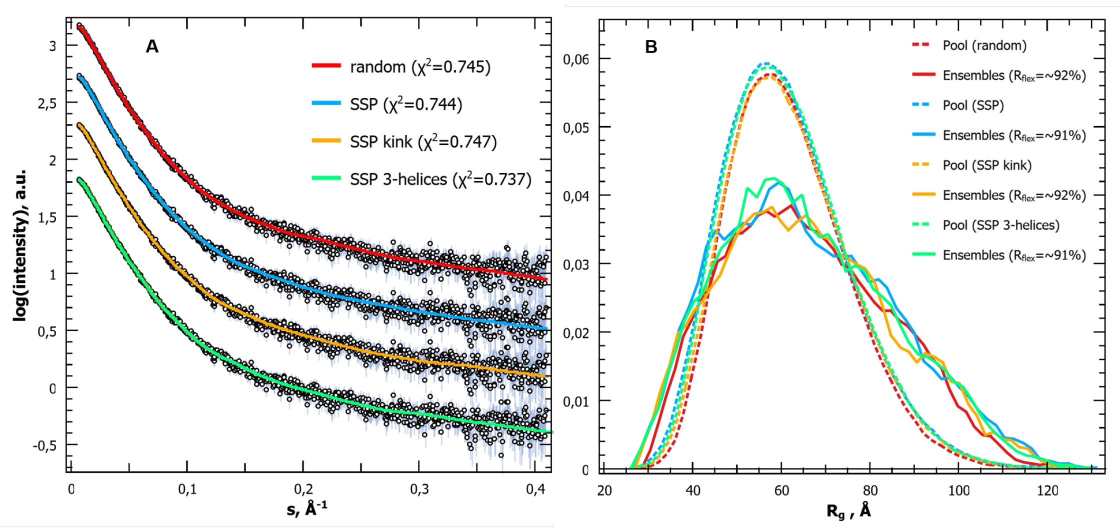

Supplementary Figure S4. **Modeling NiV PNT as a conformational ensemble. (A)** Experimental scattering curve of NiV PNT and EOM 2.0 ensemble fits obtained using different initial pools as explained in **(B). (B)** R_g distributions of the initial pools generated by: *(red dot)* EOM 2.0 (without restraints), *(blue dot)* Flexible-Meccano (with NMR secondary structure restraints), *(orange dot)* Flexible-Meccano (while imposing a kink centered at residue 20 providing NMR restraints), and *(green dot)* Flexible-Meccano (with α-helices imposed to residues 1-30). R_g distributions of the corresponding selected ensembles obtained using EOM 2.0 are shown as solid lines.

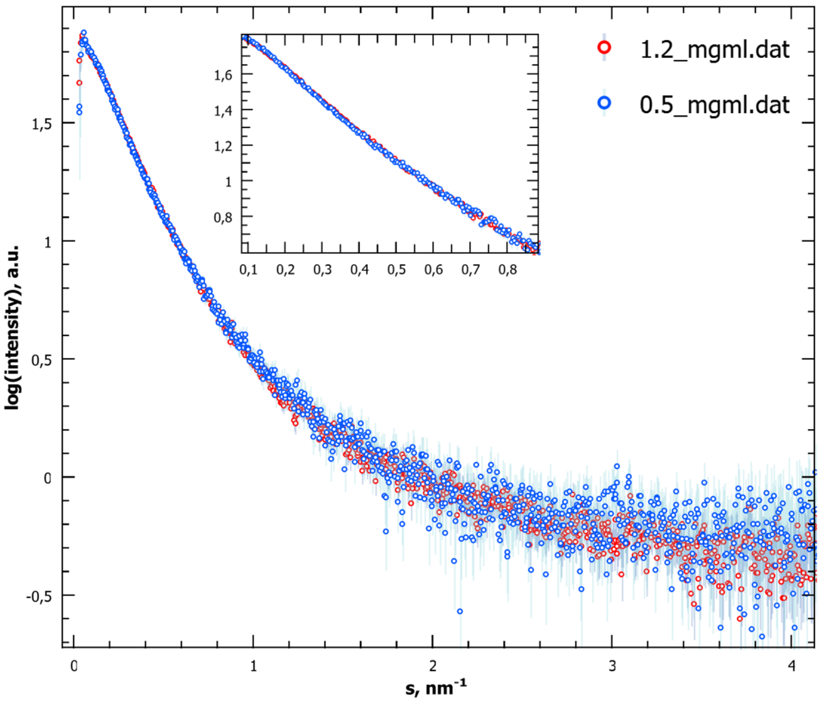

Supplementary Figure S5. Superimposition of the experimental scattering curves of NiV PNT collected at 0.5 mg/mL (*light blue*) and 1.2 mg/mL (*orange*). Inset: zoom on the superimposition at very low angle.

Supplementary Table S1. Acquisition parameters.

Experiments	Dimension of acquired data			Spectral width (ppm)			NS[a]	d1 (s)[b]
	t_1	t_2	t_3	F_1	F_2	F_3		
**^{1}H detected**								
^{1}H-^{15}N BEST-TROSY	1024 (^{15}N)	8192 (^{1}H)		35	15		2	0.05
BT HNCO	224 (^{13}C)	112 (^{15}N)	4096(^{1}H)	6	24	12	4	0.13
BT HN(CA)CO	224 (^{13}C)	112 (^{15}N)	4096(^{1}H)	6	24	12	8	0.13
BT HNCACB	148 (^{13}C)	128 (^{15}N)	4096(^{1}H)	34	25	12	4	0.20
BT HN(CO)CACB	220 (^{13}C)	128 (^{15}N)	4096(^{1}H)	65	25	12	16	0.20
BT (H)N(COCA)NNH	134 (^{15}N)	134 (^{15}N)	4096(^{1}H)	25	25	12	8	0.20
BT (H)N(CA)NNH	148 (^{15}N)	128 (^{15}N)	4096(^{1}H)	25	25	12	16	0.20
**^{13}C detected**								
CON	1264 (^{15}N)	1024 (^{13}C)		40	30		8	2.00
(H)CBCACON	164 (^{13}C)	128 (^{15}N)	1024 (^{13}C)	69	34	30	4	0.80
(H)CBCANCO	164 (^{13}C)	128(^{15}N)	1024 (^{13}C)	69	34	30	8	0.80
(HCA)COCON	108 (^{13}C)	128 (^{15}N)	1024 (^{13}C)	12	35	30	8	1.6

[a] number of acquired scans

[b] relaxation delay in seconds

Supplementary Table S2. Chemical shift (ppm) values for assigned residues of NiV PNT (aa 1-406) in 10 mM sodium phosphate buffer, pH 6.5, supplemented with 5 mM EDTA and 5 mM DTT at 288 K. ^{1}H resonances were calibrated with respect to the signal of 2,2-dimethylsilapentane-5-sulfonic acid (DSS). ^{13}C chemical shifts were referred to external DSS. ^{15}N chemical shifts were referred indirectly to the ^{13}C standard using the conversion factor derived from the ratio of NMR frequencies [9].

Number	Type	H^N	N	C^α	C^β	C′
2	ASP			54.28	41.361	175.708
3	LYS	8.442	122.574	56.413	32.901	176.375
4	LEU	8.364	124.439	55.136	42.051	177.295
5	GLU	8.389	122.708	56.187	30.119	176.175
6	LEU	8.389	123.935	54.96	42.217	177.281
7	VAL	8.147	121.906	62.253	32.653	175.97
8	ASN	8.566	123.424	52.81	38.905	174.984
9	ASP	8.354	121.881	54.531	40.954	176.829
10	GLY	8.351	109.211	45.541	//	174.413
11	LEU	8.024	121.695	55.304	42.398	177.189
12	ASN	8.523	120.187	53.115	38.681	175.206
13	ILE	8.116	122.14	61.464	37.802	176.434
28	TYR			57.809	38.751	176.383
29	GLY	8.33	110.865	45.188	//	174.11
30	ARG	8.189	121.072	55.965	30.725	176.561
31	SER	8.447	117.458	58.295	63.755	174.65
32	SER	8.365	118.503	58.114	63.675	174.439
33	ILE	8.075	122.448	61.065	38.431	176.188
34	GLN	8.381	124.958	55.45	29.448	175.584
35	GLN	8.441	123.814	53.63	28.727	173.924
36	PRO	//	137.762	63.004	31.969	176.799
37	SER	8.512	116.99	58.12	63.879	174.891
38	ILE	8.25	123.527	61.297	38.488	176.108
39	LYS	8.233	125.953	55.974	32.744	175.928
40	ASP	8.19	124.36	53.578	41.887	174.951
50	GLN			55.797	28.988	176.216
51	CYS	8.251	120.433	58.71	28.988	175.145
52	THR	8.243	116.528	61.724	69.66	174.744
53	SER	8.322	118.652	58.433	63.755	175.115
54	GLY	8.434	111.549	45.136	//	174.328
55	GLU	8.301	121.182	56.599	30.209	176.802
56	SER	8.388	117.207	58.48	63.82	174.581
57	GLU	8.442	123.256	56.548	30.092	176.422
58	GLN	8.396	122.26	55.494	29.182	175.944
59	VAL	8.244	122.538	62.016	32.775	176.366
60	GLU	8.612	125.912	56.769	29.974	177.126
61	GLY	8.559	111.586	45.352	//	174.837
62	GLY	8.304	109.203	45.208	//	174.204

63	MET	8.262	120.25	55.294	32.929	176.391
64	SER	8.467	118.256	58.236	63.705	174.638
65	LYS	8.489	124.246	56.245	33.084	182.359
66	ASP		122.234	54.163	41.216	176.104
67	ASP	8.299	122.051	54.539	40.991	177.092
68	GLY	8.433	109.548	45.52	//	174.322
69	ASP	8.233	121.339	54.441	40.969	176.87
70	VAL	8.012	120.993	63.215	32.26	176.789
71	GLU	8.385	123.271	57.19	29.627	177.111
72	ARG	8.184	122.315	56.583	30.127	182.756
73	ARG		122.199	56.473	30.554	176.481
74	ASN	8.468	119.99	53.402	38.337	175.743
75	LEU	8.209	122.931	55.915	41.952	177.977
76	GLU	8.298	121.069	56.82	30.046	176.498
77	ASP	8.241	121.499	54.525	41.028	176.729
78	LEU	8.254	123.868	55.469	41.971	178.059
79	SER	8.317	116.578	59.127	63.656	174.955
80	SER	8.211	117.849	58.34	63.375	174.85
81	THR	8.111	115.844	61.47	69.597	174.487
82	SER	8.332	120.306	56.297	63.398	172.887
83	PRO	//	138.777	63.239	32.057	177.483
84	THR	8.273	113.976	61.61	69.423	174.658
85	ASP	8.226	123.041	54.233	41.078	176.925
86	GLY	8.425	110.271	45.575	//	174.758
87	THR	8.162	114.355	62.251	69.569	175.008
88	ILE	8.102	123.563	61.341	38.479	176.862
89	GLY	8.465	113.381	45.229	//	173.91
90	LYS	8.093	121.528	55.885	33.148	176.506
91	ARG	8.413	123.779	55.903	30.882	176.346
92	VAL	8.312	122.73	62.031	32.444	176.147
93	SER	8.425	120.081	57.842	63.784	174.248
94	ASN	8.562	121.952	53.026	38.694	175.427
95	THR	8.141	114.825	61.859	69.497	174.541
96	ARG	8.297	123.397	55.901	30.52	175.879
97	ASP	8.276	122.047	54.167	40.873	176.008
98	TRP	7.982	122.191	57.283	29.406	175.815
99	ALA	7.981	126.156	52.146	19.395	177.258
100	GLU	8.132	120.87	56.836	30.019	177.123
101	GLY	8.441	111.21	45.364	//	174.334
102	SER	8.133	115.975	58.149	63.729	174.402
103	ASP		122.637	54.237	41.132	175.987
104	ASP		121.228	54.21	40.965	176.102
105	ILE	7.986	121.751	61.046	38.75	176.063
106	GLN	8.446	125.596	55.073	29.221	181.626
107	LEU		125.209	54.615	42.472	176.888
108	ASP	8.358	123.461	52.441	40.517	174.337

109	PRO	//	136.753	62.929	31.973	176.774
110	VAL	8.235	121.564	62.47	32.349	176.515
111	VAL	8.291	125.514	62.194	32.596	176.31
112	THR	8.219	118.719	61.523	69.76	174.079
113	ASP	8.291	123.727	54.184	41.058	175.988
114	VAL	8.063	121.424	62.286	32.505	175.825
115	VAL	8.093	124.952	61.953	32.665	175.534
116	TYR	8.332	125.489	57.716	38.686	175.382
117	HIS	8.18	122.235	54.912	29.933	173.766
118	ASP		122.463	54.197	40.977	176.189
119	HIS		120.293	55.715	29.092	175.43
120	GLY		110.422	45.644	//	174.805
121	GLY	8.337	109.628	45.128	//	174.298
122	GLU	8.378	121.141	56.562	30.035	176.717
123	CYS	8.495	120.86	58.416	27.696	174.899
124	THR	8.195	116.622	61.906	69.593	174.995
125	GLY	8.323	111.484	45.006	//	173.84
126	TYR	8.107	120.694	58.131	38.632	176.408
127	GLY	8.298	111.183	45.086	//	173.776
128	PHE	8.004	120.424	57.757	39.569	176.018
129	THR	8.134	116.324	61.441	69.839	174.074
130	SER	8.271	118.775	57.984	63.801	174.043
131	SER	8.321	119.125	57.208	63.365	172.969
132	PRO	//	138.58	63.552	31.971	177.151
133	GLU		121.107	56.692	29.715	176.739
134	ARG		121.976	56.188	30.518	176.75
135	GLY	8.345	110.23	45.062	//	173.987
136	TRP	8.044	121.536	57.453	29.54	176.496
137	SER	8.047	117.617	58.185	63.917	173.765
138	ASP	8.092	122.307	54.197	40.889	176.225
139	TYR	8.118	121.266	58.319	38.378	176.408
140	THR	8.029	115.609	61.747	69.603	174.747
141	SER	8.205	118.459	58.505	63.51	175.212
142	GLY	8.32	111.424	45.369	//	174.075
143	ALA	8.091	124.024	52.638	19.115	177.698
144	ASN	8.421	117.972	53.21	38.677	175.158
145	ASN	8.329	119.571	53.112	38.572	175.775
146	GLY	8.371	109.267	45.449	//	174.003
147	ASN	8.237	119.241	53.204	38.857	175.255
148	VAL	8.081	120.933	62.161	32.721	175.884
149	CYS	8.468	124.257	58.321	27.867	174.393
150	LEU	8.423	126.314	55.016	42.172	177.312
151	VAL	8.158	121.967	62.078	32.816	176.156
152	SER	8.361	120.016	58.232	63.87	174.325
153	ASP	8.281	123.517	54.108	41.092	176.307
154	ALA	8.244	125.15	53.019	18.777	178.278

155	LYS	8.258	120.081	56.431	32.591	176.994
156	MET		120.976	55.322	32.37	176.359
157	LEU	8.138	123.547	55.142	42.129	177.225
158	SER	8.1	116.395	58.113	63.737	173.799
159	TYR	8.076	122.731	57.409	38.85	174.841
160	ALA	8.129	128.213	49.979	18.424	174.8
161	PRO	//	135.873	62.732	32.018	176.89
162	GLU	8.544	121.5	56.592	30.003	176.534
163	ILE	8.147	123.078	60.825	38.609	175.797
164	ALA	8.368	129.433	52.284	19.142	177.495
165	VAL	8.157	120.831	61.982	32.787	176.286
166	SER	8.52	121.029	57.819	63.919	174.625
167	LYS	8.541	124.676	56.553	32.953	182.756
168	GLU		122.028	56.938	29.824	176.417
169	ASP	8.333	122.327	54.431	40.982	176.362
170	ARG	8.246	121.621	56.839	30.41	176.738
171	GLU	8.484	122.042	56.968	29.889	177.057
172	THR	8.073	114.959	62.256	69.552	174.429
173	ASP	8.304	123.177	54.472	40.945	176.351
174	LEU	8.116	122.536	55.469	42.151	177.748
175	VAL	7.939	120.558	62.556	32.457	182.359
176	HIS		122.481	56.173	30.088	176.182
177	LEU	8.227	123.961	55.406	42.204	177.409
178	GLU	8.499	134.621	56.554	29.971	176.395
179	ASN	8.405	120.443	53.228	38.705	175.348
180	LYS	8.294	122.531	56.413	32.76	176.739
181	LEU	8.253	123.128	55.14	42.102	177.676
182	SER	8.3	116.992	58.095	63.522	175.206
183	THR	8.252	116.02	61.556	69.29	175.067
184	THR	8.101	116.057	62.007	69.558	175.195
185	GLY	8.38	111.739	45.067	//	173.865
186	LEU	8.057	121.71	54.623	42.355	176.968
187	ASN	8.527	121.172	51.204	38.689	173.492
188	PRO	//	137.262	63.39	32.134	177.22
189	THR	8.162	114.097	61.619	69.427	174.239
190	ALA	8.145	127.195	52.154	19.266	177.361
191	VAL	8.139	122.043	59.75	32.395	174.666
192	PRO	//	139.927	62.868	32.052	
193	PHE			58.154	39.197	175.924
194	THR	7.947	116.593	61.534	69.813	173.907
195	LEU	8.123	124.993	55	42.132	177.167
196	ARG		122.315	55.824	30.569	175.788
197	ASN	8.483	120.498	52.924	38.577	175.346
198	LEU	8.306	123.645	55.305	41.896	177.464
199	SER	8.203	116.281	58.297	63.768	173.83
200	ASP	8.227	123.903	52.066	41.146	174.637

201	PRO	//	138.145	63.541	32.057	177.106
202	ALA	8.36	123.379	52.362	18.723	178.252
203	LYS	8.041	120.228	56.301	32.941	176.411
204	ASP	8.206	121.126	54.148	41.014	175.918
205	SER	8.128	117.61	56.399	63.239	172.63
206	PRO	//	138.517	62.91	31.969	176.778
207	VAL		121.35	62.453	32.429	176.308
208	ILE	8.242	126.511	60.554	38.278	175.849
209	ALA	8.36	129.238	52.332	19.222	177.484
210	GLU	8.321	120.645	56.528	30.149	176.248
211	HIS		119.796	55.277	29.83	174.076
212	TYR	8.12	122.285	57.706	38.72	175.408
213	TYR	8.227	123.021	57.803	38.661	176.068
214	GLY	7.733	109.975	45.072	//	173.918
215	LEU	8.105	121.831	55.214	42.176	178.114
216	GLY	8.497	110.534	45.13	//	174.137
217	VAL	7.925	120.037	62.28	32.502	176.389
218	LYS	8.42	125.601	56.192	32.93	176.609
219	GLU	8.42	122.815	56.832	30.151	176.342
220	GLN	8.384	121.653	55.771	29.441	175.633
221	ASN	8.539	120.656	53.27	38.805	174.975
222	VAL	8.118	120.234	62.007	32.77	176.279
223	GLY	8.337	112.992	44.404	//	171.87
224	PRO	//	134.827	63.163	31.958	177.368
225	GLN	8.643	121.26	55.782	29.262	176.419
226	THR	8.19	115.827	61.866	69.78	174.662
227	SER	8.355	118.693	58.325	63.645	174.438
228	ARG	8.366	123.369	55.907	30.727	175.904
229	ASN	8.481	120.856	53.055	38.566	175.292
230	VAL	8.11	120.821	62.247	32.704	175.725
231	ASN	8.485	122.351	53.13	38.61	175.54
232	LEU	8.284	123.655	55.533	42.087	177.467
233	ASP		120.594	54.73	40.903	176.556
234	SER	8.039	115.772	58.502	63.843	174.554
235	ILE	7.957	122.749	61.256	38.502	176.443
236	LYS		125.929	56.171	32.783	175.947
237	LEU		124.132	54.907	42.494	176.799
238	TYR	8.189	121.293	57.449	38.949	175.831
239	THR	8.178	116.371	61.064	70.108	174.297
240	SER	8.372	118.267	58.283	63.864	174.407
241	ASP	8.446	122.59	54.318	41.053	175.912
242	ASP	8.216	120.929	54.193	40.898	176.093
243	GLU		115.527	56.736	30.163	176.859
244	GLU	8.362	121.914	56.55	29.963	176.555
245	ALA	8.194	125.165	52.662	19.224	177.8
246	ASP	8.295	119.971	54.435	40.834	176.377

247	GLN	8.237	120.773	55.638	29.159	175.973
248	LEU	8.219	123.532	55.381	42.183	175.98
249	GLU		121.615	56.592	30.067	176.245
250	PHE	8.12	120.831	57.649	39.701	176.345
251	GLU		123.087	56.241	30.433	175.879
252	ASP	8.36	122.361	54.18	41.256	176.558
253	GLU	8.494	122.68	57.046	29.876	176.421
254	PHE	8.299	120.735	57.481	38.972	175.876
255	ALA	8.049	126.075	52.907	19.009	178.168
256	GLY	8.013	108.329	45.283	//	174.31
257	SER	8.199	116.132	58.444	64.075	174.836
258	SER	8.469	118.473	58.391	63.839	174.702
259	SER	8.359	118.399	58.316	63.747	174.362
260	GLU	8.345	123.4	56.413	30.456	176.191
261	VAL	8.19	122.998	62.454	32.674	175.871
262	ILE	8.361	127.308	60.648	38.301	176.184
263	VAL	8.348	126.627	62.079	32.587	176.428
264	GLY	8.466	113.609	45.007	//	173.625
265	ILE	8.038	120.671	60.661	39.021	176.301
266	SER	8.644	122.987	56.287	63.387	172.987
267	PRO	//	138.655	63.565	31.962	177.398
268	GLU	8.536	120.422	56.898	29.787	176.473
269	ASP	8.185	121.131	54.48	41.265	176.1
270	GLU	8.103	121.375	56.026	30.788	176.267
271	GLU	8.46	124.449	54.247	29.68	174.751
272	PRO	//	138.513	63.119	31.871	177.237
273	SER	8.505	116.413	58.255	63.576	174.978
274	SER	8.367	118.604	58.161	63.639	174.779
275	VAL	8.135	121.976	62.438	32.359	176.93
276	GLY	8.5	113.043	45.147	//	174.69
277	GLY	8.219	109.11	45.031	//	173.825
278	LYS	8.305	122.451	53.918	32.54	174.397
279	PRO	//	137.988	63.078	31.978	176.878
280	ASN	8.6	119.42	53.381	38.541	175.537
281	GLU	8.404	122.217	56.565	30.215	176.547
282	SER	8.383	117.274	58.352	63.567	174.74
283	ILE	8.062	123.031	61.51	38.573	176.892
284	GLY	8.49	113.129	45.253	//	174.012
285	ARG	8.123	121.187	55.895	31.165	176.542
286	THR	8.325	117.044	61.922	69.972	174.632
287	ILE	8.352	124.31	61.107	38.227	176.442
288	GLU	8.567	125.934	56.889	29.936	177.205
289	GLY	8.497	110.783	45.439	//	174.398
290	GLN	8.173	120.418	55.934	29.487	176.185
291	SER	8.456	117.814	58.628	63.564	174.811
292	ILE	8.183	123.425	61.611	38.451	176.599

293	ARG	8.296	124.815	56.477	30.804	176.301
294	ASP	8.266	121.597	54.505	40.947	176.235
295	ASN	8.324	119.432	53.37	38.471	175.657
296	LEU	8.222	122.403	55.598	41.725	177.737
297	GLN	8.221	120.662	55.717	29.142	175.956
298	ALA	8.209	125.683	52.578	19.025	177.973
299	LYS	8.268	120.942	56.287	32.899	176.389
300	ASP	8.279	121.454	53.914	41.073	175.989
301	ASN	8.332	120.182	53.089	38.627	175.386
302	LYS	8.347	122.053	56.266	32.864	176.951
303	SER	8.374	117.573	58.363	63.514	174.965
304	THR	8.181	115.721	61.521	69.42	174.268
305	ASP	8.275	123.777	54.133	40.935	175.82
306	VAL	8.145	122.636	59.954	32.581	174.563
307	PRO	//	140.658	63.311	31.913	177.724
308	GLY	8.545	110.626	45.027	//	173.927
309	ALA	8.13	124.251	52.218	19.635	178.011
310	GLY	8.341	109.241	44.396	//	171.878
311	PRO	//	135.131	62.888	32.075	177.451
312	LYS	8.53	122.165	56.075	32.816	176.624
313	ASP	8.329	122.028	54.255	41.265	176.374
314	SER	8.249	117.02	58.483	63.601	174.31
315	ALA	8.307	126.509	52.637	19.096	177.747
316	VAL	8.048	120.444	62.144	32.318	176.096
317	LYS	8.424	126.83	55.866	33.026	176.178
318	GLU		123.828	55.971	30.518	176.086
319	GLU		125.158	54.116	29.75	174.056
320	PRO	//	139.197	61.246	30.654	174.812
321	PRO	//	135.805	62.852	32.126	176.885
322	GLN		121.633	55.777	29.431	176.061
323	LYS		123.688	56.06	33.064	176.194
324	ARG		123.635	55.638	30.928	175.959
325	LEU		126.166	55.409	41.284	174.872
326	PRO	//	138.636	63.001	31.817	176.668
327	MET	8.395	120.954	55.478	33.111	176.129
328	LEU	8.299	124.244	54.885	42.33	177.107
329	ALA	8.338	125.304	52.773	19.083	177.961
330	GLU	8.427	120.055	56.837	30.035	176.641
331	GLU	8.293	121.607	57.258	29.999	176.252
332	PHE	8.293	120.807	57.511	39.718	175.732
333	GLU	8.318	122.971	56.416	30.171	176.216
334	CYS	8.418	121.018	58.283	27.958	174.819
335	SER	8.575	119.309	58.65	63.71	175.203
336	GLY	8.526	111.966	45.252	//	174.29
337	SER	8.183	115.84	58.538	63.919	174.546
338	GLU	8.51	123.099	56.333	30.239	175.924

339	ASP	8.329	124.294	52.309	41.455	174.957
340	PRO	//	137.86	63.873	32.145	177.714
341	ILE		120.437	62.092	32.785	
347	LYS			57.066	32.344	177.426
348	GLU		121.531	57.156	29.793	176.922
349	ASN	8.381	119.576	53.578	38.587	175.692
350	SER	8.201	116.632	59.077	63.517	174.87
351	LEU	8.15	123.758	55.57	41.94	177.8
352	ILE	8.15	120.115	61.369	38.281	176.266
353	ASN	8.375	122.042	53.274	38.504	175.416
354	CYS	8.247	119.98	58.757	27.778	174.831
355	GLN	8.463	122.809	56.061	29.038	176.025
356	GLN		121.704	56.073	29.413	176.554
357	GLY		110.906	45.12	//	176.553
358	LYS		121.369	56.494	32.831	176.598
359	ASP		121.099	54.189	40.889	175.785
360	ALA		124.36	52.205	19.143	177.426
361	GLN		121.178	53.258	28.868	173.577
362	PRO	//	139.123	61.159	30.659	174.715
363	PRO	//	135.238	61.311	30.542	
367	SER			58.121	63.607	174.359
368	ILE	8.16	122.844	60.766	38.684	176.047
369	GLU	8.394	121.674	56.045	29.129	
371	SER			61.897	70.026	174.335
372	ILE		122.611	60.71	38.706	176.069
373	SER		121.749	55.827	62.993	173.044
374	PRO	//	138.261	63.266	31.913	176.633
375	ASP		120.308	54.029	40.826	176.37
376	LYS	8.26	122.211	56.16	32.668	176.704
377	THR	8.18	116.072	62.234	69.633	174.497
378	GLU	8.417	124.137	56.251	30.283	176.112
379	ILE	8.244	123.608	60.887	38.202	176.38
380	VAL	8.32	126.034	62.061	32.675	175.995
381	ASN	8.664	123.963	53.346	38.59	175.767
382	GLY	8.402	109.942	45.222	//	173.745
383	ALA	8.064	124.1	52.191	19.337	177.781
384	VAL		120.325	62.272	32.571	176.375
385	GLN	8.578	125.355	55.493	29.494	176.174
386	THR	8.238	116.61	61.775	69.659	174.576
387	ALA	8.407	126.586	52.64	19.048	177.603
388	ASP	8.304	119.877	54.336	40.89	176.435
389	ARG	8.171	121.551	56.063	30.367	176.272
390	GLN	8.344	121.252	55.626	29.136	175.817
391	ARG	8.359	124.111	53.946	29.925	174.248
392	PRO	//	138.018	63.215	32.101	177.458
393	GLY	8.561	110.186	44.986	//	174.042

394	THR	8.033	117.341	59.909	69.96	172.713
395	PRO	//	139.659	62.975	32.109	176.659
396	MET	8.315	121.560	54.025	32.318	174.806
397	PRO	//	137.716	63.258	32.026	
398	LYS			56.289	32.818	176.829
399	SER	8.355	117.483	58.222	63.698	174.563
400	ARG	8.487	123.745	56.05	30.658	176.627
401	GLY	8.393	110.215	44.887	//	
402	ILE			52.868	41.367	175.221
403	PRO	//	136.107	62.932	31.897	

Supplementary Table S3. Predicted Short Linear Motifs (SLiMs) with a conservation score ≥ 0.88 as provided by the ELM database (see also **Supplementary Figure S3B**). The full report is available at:

http://elm.eu.org/cgimodel.py?fun=smartResult&userId=QiKRrpLlDz&EXPECT_CUTOFF=100&r=1&bg=on

SLiM Code	Type	Residues	Conservation Score
LIG_WD40_WDRS_VDV_2	DKEL	2-6	1.00
MOD_GSK3_1	IQKTYGRS	24-31	1.00
MOD_CK2_1	GFTSSPE	127-133	1.00
MOD_SUMO_rev_2	SDAKML	152-157	1.00
LIG_WD40_WDRS_VDV_2	TTGLNPTA	183-190	0.97
DOC_WW_Pin1_4	AKDSPY	202-207	1.00
MOD_Pro_Dkin_1	AKDSPVI	202-208	1.00
DOC_USP7_MATH_1	AGDSSS	255-259	1.00
DOC_PP2A_B56_1	LPMLAEE	325-331	1.00
CLV_PCSK_SKI1_1	RELLK	343-347	0.90
TRG_Pf_PMV_PEXEL_1	RELLKE	343-348	0.90
MOD_GSK3_1	YHWSIERS	364-371	0.88
MOD_PIK_1	IERSISP	368-374	0.97
DOC_WW_Pin1_4	RSISPD	370-375	1.00
MOD_Pro_Dkin_1	RSISPDK	370-376	1.00
LIG_FHA_1	DKTEIVN	375-381	0.88
LIG_SH3_3	RQRPGTP	389-395	1.00
DOC_WW_Pin1_4	RPGTPM	391-395	1.00
MOD_DYRK1A_RPXSP_1	RPGTP	391-395	1.00
MOD_Pro_Dkin_1	RPGTPMP	391-397	1.00
MOD_CDK_CPxxK_3	RPGTPMPK	391-398	1.00
DOC_CKS1_1	PGTPMP	392-397	1.00

Supplementary Table S4. SAXS data-collection and scattering-derived parameters.

**Data-collection parameters**	
Data source and instrument (beamline)	ESRF (BM29)
Instrument (detector)	PILATUS 1M
Beam geometry (mm^2)	0.7 x 0.7
Wavelength (Å)	0.992
Detector distance (m)	2.847
s range (nm^{-1})	0.028-4.525
Exposure time (sec)	10 (10 frames x 1.00sec)
Concentration (mg ml^{-1})	1.2
Temperature (K)	293
**Structural parameters**	
$I(0)$ (cm^{-1}) [from $P(r)$]	69.53±0.1
R_g (nm) [from $P(r)$]	6.36±0.2
$I(0)$ (cm^{-1}) [from Guinier approximation]	69.62±0.7
R_g (nm) [from Guinier approximation]	6.17±0.12
D_{max} (nm)	23±1
**Molecular-mass determination**	
Molecular mass M_r (kDa) [from *water calibration*]	~47
Calculated monomeric M_r (kDa) [from *sequence*]*	~45
**Software employed**	
Primary data reduction	PIPELINE
Data processing	ATSAS 3.0.1
Flexibility assessment	EOM 2.0

* as obtained using the ProtParam tool of the expasy server (https://web.expasy.org/protparam/)

References

1 Cordeiro, T.N., Herranz-Trillo, F., Urbanek, A., Estaña, A., Cortés, J., Sibille, N. & Bernadó, P. Structural characterization of highly flexible proteins by small-angle scattering. Adv. Exp. Med. Biol. 1009,107-129, (2017).

2 Riback, J.A., Bowman, M.A., Zmyslowski, A.M., Knoverek, C.R., Jumper, J.M., Hinshaw, J.R., Kaye, E.B., Freed, K.F., Clark, P.L. & Sosnick, T.R. Innovative scattering analysis shows that hydrophobic disordered proteins are expanded in water Science 358, 238-241, (2017).

3 Hwang, T. L., van Zijl, P. C. & Mori, S. Accurate quantitation of water-amide proton exchange rates using the phase-modulated CLEAN chemical EXchange (CLEANEX-PM) approach with a Fast-HSQC (FHSQC) detection scheme. J. Biomol. NMR 11, 221-226, (1998).

4 Piovesan, D., Walsh, I., Minervini, G. & Tosatto, S. C. E. FELLS: fast estimator of latent local structure. Bioinformatics 33, 1889-1891, (2017).

5 Callebaut, I. *et al.* Deciphering protein sequence information through hydrophobic cluster analysis (HCA): current status and perspectives. Cell. Mol. Life Sci. 53, 621-645., (1997).

6 Lieutaud, P., Canard, B. & Longhi, S. MeDor: a metaserver for predicting protein disorder. BMC Genomics 9, S25, (2008).

7 Disfani, F. M. *et al.* MoRFpred, a computational tool for sequence-based prediction and characterization of short disorder-to-order transitioning binding regions in proteins. Bioinformatics 28, i75-83, (2012).

8 Jones, D. T. Protein secondary structure prediction based on position-specific scoring matrices. J. Mol. Biol. 292, 195-202, (1999).

9 Markley, J. L. *et al.* Recommendations for the presentation of NMR structures of proteins and nucleic acids--IUPAC-IUBMB-IUPAB Inter-Union Task Group on the standardization of data bases of protein and nucleic acid structures determined by NMR spectroscopy. Eur. J. Biochem. 256, 1-15, (1998).
